# Spiritual Care in Serious Illness: A Narrative Review of Low- and Middle-Income Country Evidence

**DOI:** 10.5334/aogh.5384

**Published:** 2026-09-18

**Authors:** Ryan Meachen, Junita Henry, Sabbi Lall, Tracy A. Balboni, Constantine S. Psimopoulos, Tyler J. VanderWeele, Katelyn Long

**Affiliations:** 1 School of Psychological Sciences, Te Herenga Waka–Victoria University of Wellington, Wellington, New Zealand; 2 Department of Global Health & Population, Harvard T.H. Chan School of Public Health, Harvard University, Boston, MA, USA; 3 Harvard Divinity School, 45 Francis Ave., Cambridge, MA, USA; 4 Initiative on Health, Spirituality and Religion at Harvard University, Cambridge, MA, USA; 5 Department of Radiation Oncology, Mass General Brigham, Boston, MA, USA; 6 Department of Epidemiology, Harvard T.H. Chan School of Public Health, Harvard University, Boston, MA, USA; 7 Human Flourishing Program, Harvard University, Cambridge, MA, USA

**Keywords:** spiritual care, religious coping, serious illness, LMICs, palliative care, well-being

## Abstract

*Background:* Spiritual care (SC) is a component of person-centered care in serious illness, yet most evidence derives from high-income countries (HICs). Most patients with serious illness live in low- and middle-income countries (LMICs), where religion and spirituality often play distinctive social, cultural, and clinical roles.

*Methods:* We synthesized 46 LMIC studies drawn from the Balboni et al. [1] systematic review of 371 empirical studies on spirituality in serious illness. Eligible studies were conducted in at least one LMIC, used valid spirituality measures, included ≥100 adult participants, and were rated as low or moderate risk of bias using adapted Cochrane criteria. We examined the role of spirituality in coping and outcomes, as well as the approaches used to assess and deliver SC across Asia, the Middle East and North Africa, sub-Saharan Africa, and Latin America.

*Results:* Across the 46 studies, higher spiritual well-being and positive religious coping were consistently associated with better quality of life, lower psychological distress, and stronger family outcomes. Religious or spiritual struggle was associated with poorer mental health. SC was most often delivered by family members and faith communities rather than clinicians. Of the 46 studies, 38 (83%) used HIC-developed measurement instruments. Locally developed instruments from Thailand, Iran, and Nigeria revealed culturally distinctive pathways to spiritual well-being.

*Conclusion:* Across diverse LMIC settings, SC emerges as a generalizable yet locally inflected component of person-centered serious-illness care. We propose a both/and measurement framework retaining validated global tools while integrating locally developed instruments. Health systems in LMICs should integrate SC competencies into clinical curricula while preserving partnerships with family and faith networks.

## Introduction

Spiritual care (SC) is increasingly recognized as essential to holistic healthcare and involves “care that is respectful of and responsive to individual patient preferences, needs, and values” [1–3]. While religion and spirituality have intertwined with health and healing until recent history, and robust evidence links SC to improved outcomes in serious illness, provision remains inconsistent [4–6]. Concerned about this gap, the World Health Organization has aimed to integrate spirituality and faith-based organizations into health [7, 8]. However, SC provision goals remain unmet. Balboni et al. [1] synthesized 371 serious-illness studies and concluded that 71% to 99% of patients view spirituality as necessary, spiritual needs are common (23–98%), and SC provision is associated with better quality of life and end-of-life outcomes [1]. Yet clinicians addressed spirituality in only 9% to 51% of cases. The panel recommended implementing routine spiritual assessments, clinician training, and engaging SC specialists.

The evidence base is skewed toward North America and high-income countries (HICs), yet Asia-Pacific, African, and Latin American populations frequently report that religion plays a central role in daily life [9]. Prior syntheses draw mostly on Christian-majority or secular contexts, leaving Muslim-, Hindu-, and Buddhist-majority regions and indigenous spiritual systems understudied. Between 2020 and 2022, the share of new studies from non-American populations rose to 63%, up from 33% between 2000 and 2020 [1]. As research expands globally, it is essential to examine how existing methods are applied across diverse cultural and religious settings.

This narrative synthesis of 46 low- and middle-income countries (LMICs) studies, drawn from the Balboni et al. [1] parent review, makes three contributions. First, it presents the first systematic synthesis of spirituality and serious illness restricted to LMIC settings, spanning South and Southeast Asia, the Middle East and North Africa, sub-Saharan Africa, and Latin America. Second, it documents an inverse association between national Human Development Index (HDI) and clinician-provided SC, with implications for how SC delivery may shift as health systems formalize and traditional family and faith networks recede. Third, it identifies the dominance of HIC-developed measurement instruments in the LMIC evidence base (used in 38 of 46 studies) and proposes a both/and framework that retains validated global tools for cross-cultural comparison while incorporating locally developed instruments to capture context-specific spiritual phenomena.

## Methods

### Study design

We conducted a secondary analysis of the Balboni et al. [1] systematic review of spirituality in serious illness. The parent review synthesized evidence examining spirituality in serious illness and health outcomes, using the RAND/UCLA Appropriateness Method to combine systematic evidence with multidisciplinary expert consensus. For this analysis, we restricted inclusion to studies conducted in LMICs, as classified by the World Bank [10].

### Data sources and search strategy

The parent review searched PubMed, PsycINFO, and Web of Science from January 2000 to April 2020, with an update in May 2022, using controlled vocabulary and free-text terms for serious illness, spirituality, and measurement (see parent review; [1]). Eligible articles were published in English and included: (1) adult participants (≥18 years) with serious illness (life-limiting or life-threatening condition, substantial symptom burden, or high mortality risk), (2) a sample size of ≥100 participants, (3) cohort, cross-sectional, meta-analysis, or randomized clinical trials, (4) valid spirituality measures, and (5) a low or moderate risk of bias, as assessed using adapted Cochrane criteria. Here, we further specified that studies should be conducted in, or include, at least one LMIC.

### Study selection and data extraction

Two reviewers independently selected papers from the parent review, cross-checked them against full-text articles and supplementary material, and extracted information on country, study design, R/S variables assessed, and outcome variables. One reviewer classified countries by income group and extracted religious affiliations. A third reviewer audited, extracted information from full-text articles, categorized study design and instruments by regional origin, and extracted demographic characteristics (gender, religious affiliation, etc.). Differences in extraction were resolved by discussion. Risk of bias ratings from the parent review (using adapted Cochrane criteria and including low- or moderate-risk-of-bias studies) were retained.

### Data analysis

For this narrative synthesis, we grouped studies into five thematic categories: (1) role of spirituality, (2) spiritual needs, (3) SC provision, (4) spirituality in medical decision-making, and (5) spiritual or religious interventions. The first four domains were grouped together because of their shared focus on the relational and experiential SC. Interventions were analyzed separately due to their distinct designs and evaluative aims. Within each theme, we compared findings across regions and, where possible, religious affiliation to highlight similarities and differences potentially attributable to these contexts. Due to heterogeneity of study designs, measures, and outcomes, a meta-analysis was not conducted.

### Patient and public involvement

Patients and the public were not involved in the design, conduct, reporting, or dissemination of this research. As a secondary analysis of a published systematic review, no primary data were collected from human participants.

### Ethics approval

This study is a secondary analysis of previously published literature. No primary data were collected from human participants. Ethics committee approval was therefore not required for this synthesis.

## Results

**Figure 1:**
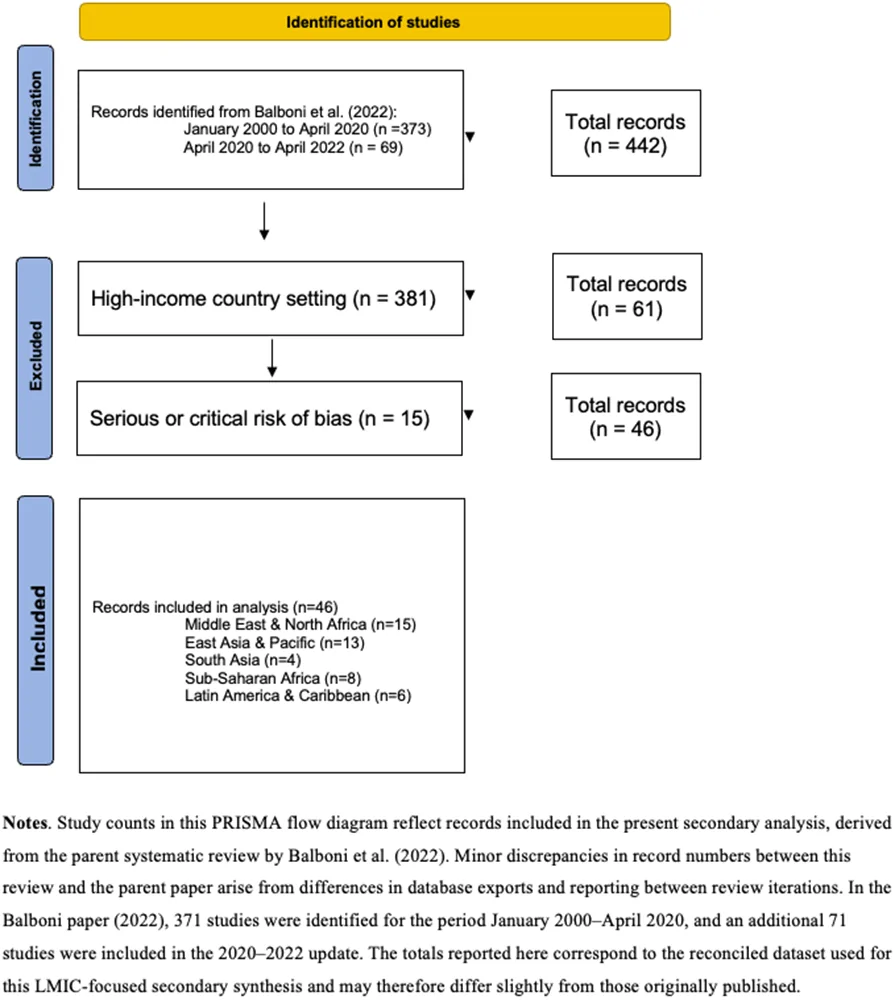
Preferred Reporting Items for Systematic Reviews and Meta-Analyses (PRISMA)-style flowchart: selection/numbers of articles for LMIC analysis.

Of the 442 serious-illness studies identified in the parent review (373 identified through April 2020 and 69 added in the 2020 to 2022 update), 61 were conducted in LMICs, with 46 studies rated as having low or moderate risk of bias (Figure 1) and 15 studies excluded for serious or critical risk of bias (Supplementary S1). 43 included studies (Supplementary S2) were patient-related, while three were caregiver-related (Supplementary S3). Therefore, we focused on patient-related insights, unless otherwise stated. The studies were partitioned into two groups: (1) Those examining the role of or need for SC, including SC in decision-making, and (2) Studies evaluating SC interventions. We also examined insights parsed by religious affiliation and the role of spirituality in medical decision-making.

## SC in Serious Illness

### Patient population

A total of 37 studies (Table 1) examined spirituality in seriously ill patients across LMICs spanning sub-Saharan Africa, the Middle East, South Asia, East and Southeast Asia, and Latin America, as well as selected upper-middle-income settings such as Brazil, China, Iran, Malaysia, South Africa, and Turkey. Most enrolled adults with advanced cancer were receiving care in hospital, palliative, or hospice settings [11–31]. Other populations included ventilated ICU survivors [32], patients with end-stage renal disease [33, 34], cardiovascular disease [35], chronic non-cancer inpatients [36], Parkinson’s disease [37], multiple sclerosis [38], HIV/AIDS [39, 40], and COVID-19 [41]. Sample sizes ranged from just over 100 to more than 1000 participants, with recruitment predominantly from tertiary hospitals, oncology units, and specialist palliative care services.

**Table 1:** SC in serious illness in LMICs (*n* = 37).

AUTHOR, YEAR	REGION; COUNTRY	RELIGIOUS OR SPIRITUAL TRADITION	POPULATION	DESIGN	MEASURES	SUMMARY OF RESULTS	OUTCOMES	BIAS RATING
Afrooz et al. [11]	Middle East; Iran	Muslim	Adult, inpatient and outpatient cancer pts recruited from Ardebil University of Medical Sciences, Ardebil, Iran	Cross-sectional survey study	Herth Hope Index (HHI); Sources of hope; Hope inspiring strategies	Of 14 sources of hope assessed, top five for pts: God 100%, prophets/imams 91%, child 67.5%, MDs 63.5%, spouse 60%. Of 20 hope-inspiring strategies assessed, top five: 97% relationship with God, 87% blessing, 84.5% controlling symptoms of disease, 82% praying, 66% appropriate manner of physician.	Description of sources of hope	Low
Akuoko et al. [12]	Sub-Saharan Africa, Ghana	Christian; Muslim	Adult female advanced breast cancer outpatients from two outpatient clinics in Kumasi, Ghana	Cross-sectional survey study	Spiritual needs (Spiritual Need Assessment for Patients, SNAP)	Most [92.1%] had at least one spiritual need (SNAP). Needs included: 92.6%, someone to bring you spiritual texts; 92.1%, finding peace of mind; 90.9%, finding forgiveness; 90.3%, your relationship with God or something beyond yourself; 90.3%, visits from clergy/imam of your own faith community; 90.3%, religious rituals such as chant, prayer, lighting candles or incense, anointing, communion; 88.6%, finding hope; 87.5%, meaning and purpose of human life; 84.7%, personal meditation or prayer practices. Older women reported greater religious needs compared to younger women.	Supportive Care Needs Survey-Long Form; modified Client Service Receipt Inventory	Low
Bar-Sela et al. [13]	Middle East (14 countries)*	Muslim; Jewish; Christian	MDs and nurses treating pts with advanced cancer	Cross-sectional survey study	Spiritual care attitudes and provision (survey adapted from the Boston Religion and Spirituality in Cancer Care study); HDI (survey developed by United Nations Development Programme: composite of life expectancy, education, per capita economic production)	Respondents were from 14 Middle Eastern countries, 40% MDs/60% nurses; 61% female; 73% Muslim; 25% very high HDI, 41% high HDI, 29% medium HDI, and 5% low HDI. Of eight spiritual care types, 42–81% considered appropriate. 47% indicated that they had provided spiritual care to any of last three advanced cancer pts. In multivariable analysis (MVA), the strongest predictor of spiritual care: staff in high-HDI countries had higher odds of not providing spiritual care than those in medium-HDI countries [AOR = 4.21 (95% CI: 2.58, 6.87)], equivalent to greater provision in medium-HDI settings]. Other significant predictors were spirituality, intrinsic religion/spirituality, training, and being a nurse. Only 22% had received spiritual care training; 77% indicated interest in receiving it.	Description of frequency and perception of importance of spiritual care provision, impact of spiritual care, and spiritual care training; Assessment of predictors of spiritual care provision	Low
Bar-Sela et al. [14]	Middle East; Middle East (14 countries*)	Muslim; Jewish; Christian	Physicians and nurses treating pts with advanced cancer	Cross-sectional survey study	Spiritual care attitudes and provision (survey adapted from the Boston Religion and Spirituality in Cancer Care study); Human Development Index (survey developed by United Nations Development Programme: composite of life expectancy, education, per capita economic production)	72% Muslim, 13% Jewish, 10% Christian. 90% at least slightly spiritual; 85% at least somewhat religious. 82% believe SC should be provided to advanced cancer pts at least occasionally, but 44% provide SC less often than they think they should. 77% had never received spiritual care training; 77% are interested in receiving training. In MVA of respondents who value spiritual care but don’t provide it to pts, predictors included low personal spirituality [*P* < 0.001] and not having received training [*P* = 0.02]. How “developed” (HDI) a country also negatively predicted spiritual care provision [*P* < 0.001]. Main perceived barriers: lack of time (66%), private space (58%), training (54%).	Description of perceptions of spiritual care; Predictors of spiritual care provision	Low
Bashar et al. [32]	Middle East; Iran	Muslim	Adult pts who had been admitted to the ICU and received mechanical ventilation in 31 provinces in Iran (45 hospitals)	Cross-sectional survey study	Pt spiritual health (Spiritual Health Questionnaire, SHQ, validated in Muslim populations)	In structural equation modeling, spiritual health (SHQ) was indirectly associated with decreased depression and anxiety [HADS: *b* = −0.081, *P* < 0.05] via pt–physician communication. Pt spiritual health had positive direct/indirect associated with greater impact of event [IES-R via PP-QoC: *b* = 0.293, *P* < 0.05], and higher post-traumatic stress scale [PTSS-14 via PP-QoC: *b* = 0.267, *P* < 0.001]. So though spiritual health is related to less depression/anxiety (indirect), SH was associated with greater post-traumatic stress disorder (PTSD) symptoms. Pt spiritual health was not related to QoL directly or indirectly.	Pt anxiety and depression (Hospital Anxiety and Depression Scale, HADS) Quality of communication (Pt– physician and Pt–nurse Quality of Communication Questionnaire) Impact of Event Scale (scale) Quality of life (Quality of Life in Mechanically Ventilated pts, QoL-MVP)	Low
Camargos et al. [15]	Latin America; Brazil	Catholic; Evangelic; Spiritist	Adult, advanced cancer pts (*n* = 525), and healthcare professionals providing direct care to oncology pts (*n* = 525) from multiple clinical settings affiliated with a tertiary care cancer center in Sao Paulo State, Brazil	Cross-sectional survey study	Assessment of perceptions of spiritual/religious care; Spiritual well-being (World Health Organization QOL: Spiritual, Religious and Personal Beliefs scale, WHOQOL-SRPB)	94.1% of pts considered it important that health professionals ask about their spiritual beliefs. Nearly all pts [99.6%] reported that spiritual support is necessary during cancer treatment; 98.3% of health professionals agreed that spiritual and religious support was necessary for oncology pts.	Description of pt and MD perceptions of spiritual/religious care; Global pt QoL (World Health Organization Quality of Life Questionnaire, WHOQOL-Brief)	Low
Chacko et al. [16]	South Asia; India	Hindu; Christian; Muslim	Pts with stage III or IV cancer being seen at a single medical center in Vellore, India	Cross-sectional survey study	Survey assessing importance of physical, emotional, social, and spiritual dimensions of end-of-life (EOL) care (modified EOL survey from Davison, 2010)	Addressing spiritual needs was moderately to very important to 96.4%. “Extremely important” aspects of spiritual care included: being able to pray [89.3%], others praying for the patient [86%], meeting spiritual advisors [63.6%], discussing spiritual issues [60.7%], and considering spiritual preferences while making decisions about life-prolonging therapies [59.3%]. Health professionals rated the same items markedly lower [52.5%, 42.5%, 42.5%, 50%, and 35% respectively]. Ideal person for addressing spiritual needs: family/friends [38.6%], spiritual advisor [35.7%], others [17.8%], nurse [5%], MD [2.9%].	Description of pt perceptions of spiritual dimensions of EOL care	Moderate
Chaiviboontham [17]	East Asia and Pacific; Thailand	Buddhist	Adult advanced cancer pts seen receiving palliative care in three hospitals in Bangkok and suburban Thailand	Cross-sectional survey study	Spiritual Well-Being Scale (SWBS) Personal Information Questionnaire (PIQ)	41.7% perceived palliative care interventions (e.g., pharmacologic and non-pharmacologic) to be effective. In MVA, spiritual well-being (SWBS) was associated with greater pt-perceived efficacy of palliative care [*b* = 0.057, SE = 0.012, *P* < 0.001].	Effectiveness of palliative care (Palliative Care Assessment Form, PCAF)	Low
Chen et al. [18]	East Asia and Pacific; China	Non-religious	Adult gynecological cancer patients recruited from a single university hospital in western China	Cross-sectional survey study	Spiritual well-being (European Organisation for Research and Treatment of Cancer spiritual well-being, EORTC QLQ-SWB32)	In MVA, global health [*β* = 0.337, *P* < 0.001] and depression [*β* = −0.144, *P* < 0.001] were significantly associated with Global-SWB.	Quality of life (European Organisation for Research and Treatment of Cancer quality of life instrument, EORTC QLQ-C30) Depression (Hospital Anxiety and Depression Scale, HADS)	Moderate
Chui et al. [42]	East Asia and Pacific; Malaysia	Muslim; Buddhism; Christian	Adult female breast cancer pts recruited from Hospital Kuala Lumpur (HKL) and the University of Malaya Medical Centre (UMMC)	Cross-sectional survey study	Complementary and alternative medicine (CAM) questionnaire, including massage, exercise, prayer for health (PFH), supplements, herbal remedies, and indigenous medicine	70.7% report using any form of CAM. The largest category of CAM-use was prayer for health [56.8% of full sample, 80.3% of CAM users], and 95.9% find prayer helpful.	Descriptive study of CAM use	Low
Dadsetan et al. [38]	Middle East; Iran		Adult multiple sclerosis patients recruited from neurology wards of Shahid Bahonar Hospital, affiliated with Kerman University of Medical Sciences in the southeast of Iran	Cross-sectional survey study	Palliative care needs including physical, social, psychological, economic, spiritual dimensions (instrument developed/validated by the authors)	All patients reported some degree of spiritual needs [*µ* = 31.2, *σ* = 4.1]. Nurses rated patients’ spiritual needs lower than patients [*µ* = 26.6, *σ* = 3.7, *P* < 0.0001]. Patient also reported physical needs [*µ* = 42.6, *σ* = 9.6], social needs [*µ* = 39.0, SD = 6.4], psychological needs [*µ* = 22.3, *σ* = 6.8], and economic needs [*µ* = 14.1, *σ* = 3.0].	Nursing assessment of patient palliative care needs (instrument developed/validated by the authors)	Moderate
Effendy et al. [19]	East Asia and Pacific; Indonesia	Muslim	Adult, hospitalized cancer (any stage) pts in three general hospitals in Indonesia	Cross-sectional survey study	Assessment of pt palliative care needs (Problems and Needs of Palliative Care scale short version, PNPC-sv)	82.4% of pts physical symptoms; 74.8% financial issues; 58.8% psychological issues; 51.3% ADLs issues; 43.7% spiritual issues; autonomy 39.5%; 27.7% social issues. 88.4% indicated spiritual issues were addressed by family [80.7%], nurses [63.4%], MDs [38.4%].	Description of symptoms and spiritual issues	Low
Farahani et al. [20]	Middle East; Iran	Muslim; Christian	Cancer MDs and nurses in government- affiliated medical facilities	Cross-sectional survey study	Assessment of spiritual care (SC) perceptions and practices (Spiritual Care Survey, adapted from the Multidimensional Measure of Religiousness and Spirituality)	70.6% of the participants considered spiritual care to be influential in the pts’ QOL. However, 64.7% had received no spiritual care training, while 82.4% indicated a willingness to receive training.	General views of spiritual care and provider perspectives on providing different types of spiritual care	Moderate
Fawares et al. [21]	Middle East; Jordan	Muslim	Adult cancer patients receiving palliative care at a single hospital in Jordan	Cross-sectional survey study	Religious beliefs and values (Arabic Beliefs and Values scale)	Patients had high endorsement of religion and spirituality; they had greater endorsement of the spirituality religious beliefs items [3.38 ± 0.33] than of the spirituality non-religious beliefs aspect [2.49 ± 0.50]. 79% had never attended a spirituality session and 74.8% had never been visited by a religious/spiritual advisor in hospital	Descriptive data only	Moderate
Gravier et al. [22]	Mixed; Brazil, Chile, India, Jordan, USA	–	Adult advanced cancer pts recruited from five outpatient palliative care clinics in Brazil (*n* = 131), Chile (*n* = 71), India (*n* = 44), Jordan (*n* = 182) and the USA (*n* = 300); total *n* = 728	Cross-sectional survey study	Meaning in life (Meaning in Life Scale, MILS); spiritual pain and financial distress (0–10 numeric scales administered alongside the Edmonton Symptom Assessment System, ESAS-SP and ESAS-FD); optimism (1–7 numeric rating scale)	Meaning in life (MILS) did not differ between the five countries [*P* = 0.11], though domain sub-scores differed. In MVA, higher meaning in life was associated with greater optimism [*b* = 0.33, *P* < 0.001], lower depression [*b* = −0.26, *P* < 0.001], less spiritual pain [*b* = −0.19, *P* < 0.001], and less financial distress [*b* = −0.16, *P* < 0.001].	Symptom burden, depression and physical symptoms (Edmonton Symptom Assessment System, ESAS); performance status (Karnofsky)	Moderate
Haokip et al. [23]	South Asia; India	Hindu	Adult cancer patients recruited from a single, large government hospital, All India Institute of Medical Sciences, Rishikesh, India	Cross-sectional survey study	Spiritual beliefs (System of Belief Inventory, SBI-15R)	Most (77%) considered themselves moderately or very spiritual. In univariable analysis, spirituality was inversely associated with depression [*r* = −0.209, *P* < 0.05].	Depression symptoms (Patient Health Questionnaire Hindi version, PHQ-9)	Moderate
Kwok et al. [37]	East Asia and Pacific; Hong Kong, Taiwan, Thailand	Buddhist; None; Christian	Adult Parkinson’s disease patients were recruited from neurological clinics in China (Hong Kong), Taiwan, and Thailand	Cross-sectional survey study	Unmet supportive care needs, including physical symptoms; emotional, psychosocial, and spiritual needs; and provision of information and practical concerns (Palliative care Outcome Scale, POS, the Chinese version for HK and Taiwan, and a Thai version for Thailand)	55.4% reported unmet spiritual needs (POS). Degree of spiritual need varied by country: Taiwan 64.1%, Hong Kong 70.3%, and Thailand 29.3% [*P* < 0.05]. Other common supportive care needs included: physical symptom needs [62.9%], psychosocial needs [64%], and anxiety needs [52.2%].	Symptom burden (Palliative Care Outcomes Symptom—Parkinson’s Disease) Quality of life (7-item EQ-5D)	Moderate
Liu et al. [36]	East Asia and Pacific; China		Adult cancer inpatients and adult non-cancer chronic illness inpatients recruited from the Chongqing University Three Gorges Hospital	Cross-sectional survey study	Spiritual well-being (Functional Assessment of Chronic Illness Therapy, FACIT-Sp, Chinese version)	97% of patients had no religious affiliation; spiritual well-being was moderate among cancer patients versus chronic illness (non-cancer) patients; and cancer patients’ SWB scores were lower than those of patients with chronic illnesses [FACIT-Sp: *µ*(cancer pts) = 33.30, *σ* = 10.35; *µ*(chronic illness pts) = 38.61, *σ* = 10.88, *P* < 0.001].	Descriptive assessment of spiritual well-being (SWB), with comparisons of SWB between those with cancer versus chronic diseases, and predictors of SWB	Moderate
Lowther et al. [39]	Sub-Saharan Africa; Kenya		Adult HIV pts, on ART for at least 1 month, with a pain or symptom score of 3–5 (from a possible range of 0-best to 5-worst) on the African Palliative Care Association Palliative Outcome Scale (APOS), being seen at an HIV clinic in Mombasa	Randomized controlled trial	Intervention: five visits to a palliative care nurse who provided assessment and care plan for addressing physical, psychological, social and spiritual problems. Complex pts were referred to specialty palliative care Controls: Usual HIV care (monthly clinic appointments) vs. usual care (1:1) *n* = 60 per arm	A RCT of a nurse-led palliative care intervention for pts with HIV found in assessments of 22 components of the palliative care intervention (medication and psychosocial), discussion about spiritual worries was higher in the intervention arm vs. usual care [95% vs. 57%, *P* < 0.001], emotional support from staff [100% vs. 80%, *P* < 0.001], use of weak opioids [60% vs. 18%, *P* < 0.001], discussion about the future [67% vs. 22%, *P* < 0.01] constipation medication [27% vs. 12%; *P* = 0.04], support for the family in planning for the future [65% vs. 22%, *P* < 0.001]. Mental well-being had greater numeric improvements in intervention arm vs. usual care [57% vs. 52%, but no analyses reported].	Care received (Client Services Receipt Inventory, CSRI); Qualitative exit interviews to further assess pt perceptions of the intervention; Mental health (MOS-HIV)	Moderate
Mah et al. [43]	Mixed; Canada, Kenya	Christian	Pts who died in three Kenyan hospices [*N* = 127], compared to pts who died of advanced cancer in Ontario, Canada [*N* = 602]	Cross-sectional survey study	Patient quality of death and dying as assessed by the family caregiver (The Quality of Death and Dying Questionnaire, QODD), with six domains: Symptoms and Personal Care, Preparation for Death, Moment of Death, Family, Treatment Preferences, and Whole Person Concerns	As compared to the Canadian sample, quality of death outcomes (QODD) in the Kenyan sample were worse on 14 quality of dying and death concerns and on overall quality of dying and death [all *P* < 0.001] but better on five concerns, including interpersonal and religious/spiritual concerns [all *P* < 0.005]. In correlational analysis, overall quality of dying was associated with better pt care experiences with symptoms and personal care, interpersonal care, and of religious/spiritual concerns [all *P* < 0.01].	Description of quality of death and dying, with comparisons between the Canadian and Kenyan samples	Moderate
Malhotra et al. [24]	Mixed; China, India, Myanmar, Sri Lanka, Vietnam		Adult advanced cancer patients recruited from seven hospitals in five countries (China, India, Myanmar, Sri Lanka, and Vietnam)	Cross-sectional survey study	Spiritual suffering (reverse scaling of the meaning/peace domain of the spiritual well-being scale to function as a spiritual suffering scale; Functional Assessment of Chronic Illness Therapy-FACIT-Sp)	Among advanced cancer patients, “Spiritual suffering” (reversed meaning/peace FACIT-Sp) is less among those with more education [*b* = −0.26, SE = 0.12, *P* < 0.05], is greater among those of lower socioeconomic status [*b* = 7.57, SE = 1.66, *P* < 0.01], and is greater in Sri Lanka [*b* = 16.55, SE = 2.04, *P* < 0.01], India [*b* = 11.53, SE = 1.89, *P* < 0.01], and Vietnam [*b* = 5.81, SE = 2.02, *P* < 0.01] as compared to China.	Physical and functional suffering (physical and functional well-being sub-scales of the Functional Assessment of Cancer Therapy-General, FACT-G version 4) Pain (Visual Analogue Pain Scale) Psychological suffering (Hospital Anxiety and Depression Scale, HADS) Social suffering (social subscale of the FACT-G)	Moderate
Martinez and Custódio [33]	Latin America; Brazil		Adult end-stage renal disease on hemodialysis pts from a single medical center in Brazil	Cross-sectional survey study	Spiritual well-being (Spiritual Well-Being Scale, SWBS)	In MVA, spiritual well-being (SWBS) predicted better mental health (GHQ) [adjusted *r*^2^ = 0.0124, *f* = 5.165, *P* = 0.0009]. Associations were driven by existential rather than religious well-being	Mental health (General Health Questionnaire, GHQ)	Moderate
Mishra et al. [44]	South Asia; India		Adult cancer inpatients and outpatients, and their informal caregivers (when present) seen by oncology and palliative care services during the COVID-19 pandemic at a single hospital in New Delhi.	Cross-sectional survey study	Spiritual suffering during the COVID-19 pandemic questionnaire (three-item questionnaire developed by the authors)	53.6% of patients reported lack of spiritual clarity and hope; among caregivers, 20% reported suffering a lack of spiritual clarity and hope. 35% of patients and 30% of caregivers reported challenges due to a lack of religious practice during the pandemic.	Other domains of suffering during cancer care in the COVID-19 pandemic were examined: physical, logistic (including treatment delays), psychological, and socioeconomic	Moderate
Myint et al. [25]	East Asia and Pacific; Myanmar	Multi-faith	Adult cancer patients (>50 y, all stages) seen at three oncology clinics in Mandalay, Myanmar	Cross-sectional survey study	Palliative care needs (Palliative care assessment tool, including psychosocial and spiritual needs)	90.1% of patients desired spiritual support. 73.8% wanted help to find meaning in their cancer experience. 85.8% were willing to receive palliative care services. In MVA, greater spiritual and psychosocial need predicted willingness to receive palliative care services [aOR = 3.76, (95% CI: 1.17, 12.05), *P* = 0.03].	Willingness to receive palliative care services; Social support (structural-functional social support scale, SFSS)	Low
Ndiok and Ncama [45]	Sub-Saharan Africa; Nigeria	–	Adult cancer pts seen at two teaching hospitals in Nigeria, cgs could respond on behalf of cognitively impaired pts	Cross-sectional survey study	Pt spiritual and existential issues (study developed scale to assess pt spiritual/existential issues for their population, with literature review, pilot testing, and validation)	Existential/spiritual needs were reported by 49.7% to 71.8%, including 71.8% difficulties with acceptance; 63.6% difficulties in feeling useful; 55.7% difficulties in being available to others; 55.0% difficulties concerning the meaning of death, 49.7% difficulties in confidence in God/religion.	Description of spiritual needs and existential issues	Low
Nkhoma et al. [40]	Sub-Saharan Africa; Kenya	–	Adult, HIV/AIDS pts recruited from an outpatient clinic in Mombasa, Kenya	Cross-sectional survey study	Existential/spiritual concerns (African Palliative Outcomes Scale, APOS)	36.8% reported being in lowest three categories of peace (APOS), 23.5% in lowest three categories of life worthwhile. In MVA, decreasing age associated with greater existential/spiritual concerns [*P* = 0.001], as was higher CD4 count [*P* = 0.014], and TB treatment [*P* = 0.02].	Description of existential and spiritual concerns	Moderate
Ramirez et al. [34]	Latin America; Brazil	Catholic; Protestant; Other Christian	Adult end-stage Renal disease (ESRD) patients from three outpatient hemodialysis units in Brazil	Cross-sectional survey study	Positive religious coping and religious struggle (Brief RCOPE)	In MVA, religious struggle correlated with both depressive [*b* = 0.31, *P* < 0.0001] and anxiety [*b* = 0. 36, *P* < 0.0001] symptoms. In MVA, positive religious coping was associated with better overall QoL [*b* = 0.17, *P* = 0.02], while religious struggle was associated with worse overall QoL [*b* = −0.26, *P* < 0.001].	Depression and anxiety (Hospital Anxiety and Depression Scale, HADS); Quality of life (the World Health Organization QoL instrument, abbreviated version, WHOQOL-Brief)	Low
Ratshikana-Moloko et al. [26]	Sub-Saharan Africa; South Africa	Christian; Ancestral beliefs; Other	Adult advanced cancer pts recruited from the Gauteng Center for Palliative Care in Soweto, South Africa	Prospective cohort study	Pt reports of religious/spiritual needs and care (Spiritual Needs and Care questionnaire); Religious/spiritual characteristics (Coping with Cancer study items)	Most pts were religious or spiritual [94.8%]. Most reported having spiritual needs [97.8%], with the most common being seeking a closer connection to God [92.6%] and forgiveness of sins [90.8%]. 39.5% of pts received spiritual care. Pts receiving spiritual care had less pain [2.82 ± 1.23 vs. 1.93 ± 1. 69] and were more likely to die at home [57.5% vs. 33.7%, *P* = 0.012]. In MVA, spiritual care associated with reduced pain [POS: OR = 0.33 (95% CI: 0.11, 0.95)] and increased family worry [POS: OR = 3.43 (95% CI: 1.10, 10.70)].	Description of pt spiritual needs and care; Pt palliative care outcomes (Palliative Care Outcomes scale, POS); Pt location of death	Moderate
Rohani et al. [27]	Middle East; Iran	–	Adult women all stages of breast cancer receiving care at 1 of 2 educational medical facilities in Iran, Tehran	Prospective cohort study (6 months)	Spiritual beliefs and behaviors (Spiritual Perspectives Scale, SPS) Religious coping (RCOPE positive and RCOPE negative) Sense of coherence/meaning to life (Sense of Coherence Scale, SOC)	In MVA, though spiritual beliefs and behaviors (SPS), and religious coping (RCOPE Pos and RCOPE neg) did not predict improvements in QOL (EORTC QLQ-C30) at 6 months, Sense of Coherence (SOC) did predict improvements in QOL at 6 month [EORTC QLQ: *b* = 0.50, *P* < 0.001].	Quality of life (European Organisation for Research and Treatment of Cancer scale, EORTC, QLQ-30)	Low
Şahan [41]	Middle East/North Africa; Turkey	–	Adults diagnosed with COVID-19 in Turkish hospitals (55% hospitalized), surveyed online (Google Forms) (*n* = 384)	Cross-sectional survey study	Spiritual needs (Spiritual Care Requirements Scale)	Spiritual needs (Spiritual Care Requirements Scale score) of the participants was 67.05 ± 26.30 (possible 21–105). The mean VAS for Death Anxiety score of the participants was 8.82 ± 1.26 (possible 0–10).	Death anxiety (Visual Analog Scale, VAS)	Moderate
Satija et al. [46]	South Asia; India	Hindu; Muslim; Other	Adult advanced cancer patients who were recruited from oncology and palliative medicine clinics at a tertiary cancer hospital in India	Cross-sectional survey study	Spiritual Well-being (FACIT-Sp)	73% were not aware of the stage of their malignancy. In MVA, patients who were aware of their cancer diagnosis had worse FACIT-Sp faith subdomain scores [*b* = −1.6, (95% CI: −3.1, −0.1, *P* = 0.03] (alternate numerical interpretation is those with greater spiritual well-being have less awareness of their cancer).	Awareness of stage of malignancy	Moderate
Selman et al. [47]	Sub-Saharan Africa; South Africa, Uganda	–	Adult cancer, HIV, and other advanced illness pts seen at four palliative care facilities in South Africa and one hospice in Uganda	Cross-sectional survey study	Quality of life (Missoula Vitas Quality of Life Index, MV-QVQOLI): subscales symptoms, function, interpersonal, well-being, transcendent; assessment of what is important to pts using QVQOLI	Pts assessed what is most important to their QOL, which were: close relationships [mean = 4.13], feeling at peace [mean = 4.12], sense of meaning in life [mean = 4.10], being active [mean = 3.84], physical comfort [mean = 2.58]. The transcendent subscale was most highly correlated with pt global QoL [*r* = 0.77, *P* < 0.001], as compared to other domains of QoL.	Description of pt quality of life and description of what pts deem as most important to quality of life	Low
Valentino et al. [28]	Latin America; Brazil	Any religion recorded as present or absent only (97.9% of pts reported having a religion); denomination not recorded	Adult patients (and adult caregivers) with advanced incurable cancer, undergoing systemic palliative treatment and/or individualized palliative care, life expectancy >3 and ≤12 months, performance status ≤3, full cognitive capacity and coherent communication skills recruited from the Barretos Cancer Hospital in Brazil.	Cross-sectional survey study	Spirituality (1 of 6 End of Life factors of importance rated by patients and caregivers)	Of six factors most important at the end-of-life, patients and caregivers reported that presence of loved ones [42.3% pts, vs. 44.2% cgs] was the factor of greatest importance, followed by spirituality [38.5% pts vs. 27.9% cgs] and the place-of-death [14.0% pts vs. 18.4% cgs].	Preferred place of death outcome (questionnaire developed/validated by the research team) End of life factors of importance (scale) Depression and anxiety (Hospital Anxiety and Depression Scale, HADS)	Moderate
Wisesrith et al. [29]	East Asia and Pacific; Thailand	Buddhist	Adult terminally ill cancer patients from seven hospitals in northern, northeast, central, and southern regions of Thailand	Cross-sectional survey study	Spiritual needs (Spiritual Needs Scale, developed and validated by the authors)	The overall spiritual needs of terminal ill cancer patients were at the moderate level [*µ* = 18.21, *σ* = 2.56]. The highest mean was found in the “prepare for death” dimension, followed by the “have meaning, values, and life purposes,” and the “have opportunity to pursue most important things in life” dimensions respectively and had different spiritual needs among married persons (versus widowed) [*F*(3,318) = 3.66, *P* < 0.05], with increasing number of family members [*F*(8,313) = 5.07, *P* < 0.05], living with family [*F*(2,319) = 3.91, *P* < 0.05], and having a spiritual anchor (key spiritual supporter) [*F*(2,319) = 4.13, *P* < 0.05].	Descriptive study of spiritual needs in Thai patients	Moderate for description of spiritual needs; Serious for predictors of spiritual needs
Yaghoobzadeh et al. [35]	Middle East; Iran	Muslim	Adult pts with cardiovascular disease hospitalized for greater than 24 hours in a medical institution in Iran	Cross-sectional survey study	Spiritual well-being (Spiritual Well-Being Scale, SWBS)	Mean spiritual well-being (SWBS) was 86.21 (SD 12.46; range 40–116) and mean hope (HHI) was 34.80 (SD 5.05; range 23–46), indicating higher than moderate spiritual well-being and a moderate level of hope. In MVA, hope was a significant predictor of spiritual well-being [*b* = 0.90, 95% CI 0.68–1.13, *P* < 0.001], as were religious belief [*b* = 0.99, 95% CI 0.17–1.80, *P* = 0.018] and female sex [*P* = 0.047]. Spiritual well-being was in turn a significant predictor of hope [*b* = 0.12, 95% CI 0.09–0.16, *P* < 0.001], alongside marital status, educational status and socioeconomic status.	Hope (Herth Hope Index, HHI)	Moderate
Yang et al. [30]	East Asia and Pacific; China	–	Adult advanced metastatic cancer patients with a life expectancy < 6 months and admitted to the hospice ward of Shengjing Hospital of China Medical University	Prospective observational study (2 weeks)	Spiritual well-being (Functional Assessment of Chronic Illness Therapy-Spiritual Well-being)	Patients’ existential well-being [*b* = −0.99, (95% CI: −1.72, −0.26), *P* = 0.008] and meaning dimension [*b* = −0.87, (95% CI: −1.29, −0.43), *P* < 0.001] significantly decreased after admission to the PC unit, but peace and faith did not change over time.	Anxiety and depression (Hospital Anxiety and Depression Scale) Pain (Numerical Rating Scale)	Moderate
Zafar et al. [31]	Middle East; Pakistan	Most (80–96%) reported that religion/religious practice plays an essential role in their lives.	Adult cancer pts undergoing treatment at the tertiary cancer center in Lahore, Pakistan	Cross-sectional survey study	Assessment of religiousness, Preferences regarding EOL care, Pt EOL priorities (survey developed for this study from validated instruments: Multidimensional Health Locus of Control scales, Singer et al. EOL care items, survey piloted)	96% reported religion as important (4% not important); most (80–96%) endorsed an important role of religion/religious practice in their lives. Average rank order preferences of priorities at EOL: (1) spiritual/religious well-being; (2) not being a burden to others; (3) strengthening relationships with family/friends; (4) receiving adequate pain control and symptoms management; (5) achieving a sense of control and dignity; (6) avoiding inappropriate prolongation of dying.	Description of pt religiousness and EOL priorities	Moderate

***Notes:*** *Cyprus, Egypt, Iran, Iraq, Israel, Jordan, Lebanon, Pakistan, Palestine, Saudi Arabia, Sudan, Oman, Turkey, United Arab Emirates.

### Study design and analysis

Most studies (89%) were cross-sectional and used structured questionnaires. Three were prospective (two advanced cancer cohorts [26, 27] also assessing receipt of SC [30]) and one used structural equation modeling to examine mediation pathways [32]. Most analyses employed multivariable linear or logistic models. Two Middle Eastern studies surveyed oncology clinicians (*n* = 770) with different outcomes [13, 14].

### Measures

A wide range of instruments was used (Supplementary material S4). Spiritual needs were measured using the Spiritual Needs Assessment for Patients (SNA [12]), the SC Requirements Scale (SCR [41]), and culturally adapted or study-specific tools, including scales developed for Thai cancer patients [29] and for multiple sclerosis in Iran [38]. In India, the FACIT-Sp was translated and validated to assess spiritual well-being in patients with advanced cancer, following a rigorous forward–backward translation and cognitive testing process [46]. Spiritual well-being was most often assessed using the Spiritual Well-Being Scale (SWBS [17, 33, 35]), the Functional Assessment of Chronic Illness Therapy–Spiritual Well-Being (FACIT-Sp [24, 30, 36]), and the EORTC QLQ-SWB32 [18].

Religious coping, beliefs, and faith constructs were examined using the Brief Religious Coping Scale (RCOPE [27, 34]), the System of Belief Inventory (SBI-15R [23]), and the Arabic Beliefs and Values Scale (BVS [21]). Faith and prayer were assessed using the Prayer for Health (PFH) items [42]. Hope and meaning/optimism were captured using the Herth Hope Index (HHI [35]), the Life Orientation Test–Revised (LOT-R [30]), and meaning-in-life scales [11, 22]. Some studies compared nurse versus patient-rated spiritual needs [38] or contrasted patient and clinician perceptions of spiritual QoL [15]. Denomination, religious affiliation, and self-rated religiosity/spirituality were routinely collected as demographic variables, as well as clinician personal spirituality/intrinsic religiosity [13, 14, 16, 23].

### Regional findings

#### Asia

Across Asia, spirituality was frequently integral to coping, end-of-life preferences, and QoL. Pakistani cancer patients ranked spirituality as their highest end-of-life priority, above physical comfort [31]. In India, during the COVID-19 pandemic, 54% of patients and 20% of caregivers reported lack of spiritual clarity and hope, with significantly higher distress among patients [44]. Most valued prayer, spiritual advisers, and family support over clinician involvement [16]. In another Indian study of 200 advanced cancer patients, 73% were unaware of their disease stage, and lower scores on the FACIT-Sp faith subdomain were associated with greater awareness of malignancy stage (*β* = −1.6; 95% CI: −3.1 to −0.1; *P* = 0.03; FACIT-Sp total not significant, *P* = 0.29) [46]. A five-country cohort (China, Sri Lanka, India, Vietnam, Myanmar) showed that patients with lower income or education experienced greater suffering, including spiritual distress [24].

In East Asia, among Chinese hospice patients, existential well-being scores declined over a two-week period (*β* = −0.99; *P* = 0.008), while peace and faith remained stable [30]. In Chinese gynecological cancer patients, greater spiritual well-being was associated with better global health and lower depression [18]. Across Hong Kong, Taiwan, and Thailand, patients with Parkinson’s disease reported moderate unmet support needs (mean Palliative Care Outcome Scale [POS] score of 10.5 ± 6.4); psychosocial and spiritual needs were prioritized in Hong Kong and Taiwan, whereas physical and emotional concerns dominated in Thailand. Younger age, male sex, and advanced disease stage were associated with a higher psychological and spiritual burden [37].

#### Middle East and North Africa

In Iran and Jordan, spiritual and religious well-being was central to comfort and hope. Iranian patients identified faith in God, the Prophet, and the Imams as key sources of hope, with higher spiritual well-being associated with greater hope and a better mood [11, 35]. Jordanian palliative patients endorsed religious spirituality more than non-religious spirituality [21]. Greater spiritual health among Iranian ICU survivors was associated with reduced depression and anxiety mediated through patient–physician quality of communication, but was paradoxically associated with higher post-traumatic stress [32]. Nurses significantly underestimated Iranian multiple-sclerosis patients’ psychological and social needs, while rating physical and economic needs higher than patients did [38]. Turkish patients hospitalized with COVID-19 had moderate-to-high spiritual-care requirements (mean SCRS 67.1 ± 26.3) and high death anxiety (VAS 8.8 ± 1.3); male patients and those with chronic illness reported higher scores for understanding and hope [41]. In Turkey, fewer than 2% of surveyed nurses were trained in SC (*N* = 1,254) [48]. Similarly, in Iran, only one-third of oncology nurses and physicians had training in SC, despite 70% agreeing that SC improves QoL. Key obstacles included a lack of time and fear of patient discomfort [20]. Two oncology surveys involving 770 physicians and nurses across 14 Middle Eastern countries revealed that 82% of staff endorsed SC, yet reported providing it to only 47% of their three most recent patients with advanced cancer, and 44% provided it less often than they believed they should [13, 14]. Additionally, medium national HDI countries provided SC more often than those in high-HDI countries (OR 4.21, 95% CI: 2.58–6.87), consistent with an inverse development gradient [13]. Lack of personal spirituality and inadequate training independently predicted non-provision of SC among staff who endorsed it.

#### Sub-Saharan Africa

Spiritual concerns were widespread and clinically relevant in sub-Saharan Africa. In Ghana, ≥85–93% of women with advanced breast cancer reported spiritual needs for scripture, clergy visits, peace, or forgiveness, with younger women expressing fewer religious needs [12]. Nigerian patients reported substantial existential distress (~50–72%) and frequent reliance on faith healing or traditional medicine, delaying hospital presentation [45]. Among Kenyan HIV/TB outpatients, older age predicted better existential well-being (adjusted OR 1.03; *P* = 0.001), while higher CD4 counts and TB treatment correlated with worse spiritual scores, suggesting a persistent need despite clinical improvement [40]. In South Africa, receiving SC correlated with lower odds of pain in adjusted analysis (adjusted OR 0.33; 95% CI: 0.11–0.95; *P* = 0.04), greater likelihood of home death (57.5% vs. 33.7%), but higher family worry (OR 3.43); nearly all patients (97.8%) reported spiritual needs, 85% were Christian, and older women were more likely to receive care [26]. In South Africa and Uganda, peace, meaning, and relationships were the strongest predictors of QoL, with the transcendent domain showing the highest correlation with total well-being (*r* = 0.77) [47]. Kenyan family caregivers rated patients’ interpersonal and religious or spiritual concerns at the end of life as well or better than their Canadian counterparts did [43].

#### Latin America

In Brazil, negative religious coping predicted poorer health-related quality of life (HRQoL) and greater anxiety and depression in hemodialysis patients. In contrast, positive coping and higher spiritual well-being were associated with better mental health and social functioning [33, 34]. At the end of life, Brazilian cancer patients prioritized the presence of loved ones (42%) and spirituality (39%) [28]. A multinational study (Brazil, Chile, India, Jordan, USA) found that meaning in life is inversely associated with spiritual pain, depression, and financial distress, and positively associated with optimism, without cross-country differences [22].

### Cross-regional summary

Across LMICs, spirituality was central to coping, QoL, and end-of-life priorities. Despite near-universal spiritual identification, unmet spiritual needs were common and strongly associated with poorer psychological, physical, and existential outcomes. In contrast, positive religious coping and higher spiritual well-being correlated with improved mood, hope, and QoL. SC delivery was more strongly correlated with system inputs (training, chaplaincy availability) and clinician attributes (personal spirituality) than with patient-expressed need, and was inversely associated with national HDI in the Middle East [13, 14].

## Spiritual Care Interventions in Serious Illness

### Patient population

Seven studies (Table 2) across Asia (Iran, China) and Africa (Kenya) examined the effects of SC interventions on individuals with serious illnesses, one meta-analytic and others mostly advanced or terminal cancer patients [49–52]. Additional populations included people living with HIV [39], family caregivers of older adults with Alzheimer’s disease [53], and patient–family dyads facing hematologic malignancies [54]. Several interventions were culturally adapted for Muslim populations [49, 53] or explicitly integrated family caregivers into the therapeutic process [30, 52]. Sample sizes ranged from 100 to 623 participants.

**Table 2:** Spiritual interventions in serious illness in LMICs (*N* = 7).

AUTHOR, YEAR	REGION, COUNTRY	RELIGIOUS OR SPIRITUAL TRADITION	POPULATION	DESIGN	INTERVENTION	MEASURES	SUMMARY OF RESULTS
Wang et al. [54]	East Asia, China	Nullifidian; Buddhist; Christian	Adult hematological malignancy patients and their informal family caregivers were recruited from Fujian Medical University Union, Fujian, China	Randomized trial (8 weeks)	Family participatory dignity therapy; Control: Treatment as usual	Patient hope (Herth hope index, HHI); Patient spiritual well-being (Functional assessment of chronic illness therapy-spiritual well-being scale, FACIT-Sp); Caregiver anxiety (self-rating anxiety scale, SAS); Caregiver depression (self-rating depression scale, SDS); Family adaptability (Family adaptability and cohesion evaluation scale-II, FACES II, for patients and family caregivers)	For patients, there were significant improvements in hope [*P* = 0.001], spiritual well-being [*P* = 0.002], and family cohesion [*P* < 0.001] and adaptability [*P* < 0.001] between the intervention and control groups. The difference over time was also significant in family cohesion [*P* = 0.018] and adaptability [*P* = 0.003]. The interaction effects were significant for hope [*P* = 0.034], spiritual well-being [*P* < 0.001], and family cohesion [*P* < 0.001] and adaptability [*P* < 0.001]. For family caregivers, there was a significant difference in anxiety [*P* = 0.037], depression [*P* = 0. 001], and family adaptability [*P* = 0.036] between the intervention and control groups. Within groups, a significant difference in family adaptability [*P* = 0.012] was found. Moreover, the interaction effects were significant on anxiety [*P* = 0.001] and family cohesion [*P* = 0.038].
Xiao et al. [52]	East Asia, China		Adult lung cancer patients receiving chemotherapy, and their informal caregivers, at a cancer hospital in Changsha, China	Randomized trial (4 weeks)	Family-oriented dignity therapy; Control: Attention (education)	Dignity (Patient Dignity Inventory), Depression (Patient Health Questionnaire-9), Spiritual well-being (Functional Assessment of Chronic Illness Therapy-Spiritual Well-being Scale, FACIT-Sp)	Patients in the intervention group showed significantly greater reduction in existential distress [*β* = −1.372, (95% CI: −2.269, −0.472), *P* = 0.003] and depression [*β* = −3.430, (95% CI: −5.032, −1.829), *P* < 0.001] at week one, as well as significantly greater improvement in spiritual well-being at both week one [*β* = 3.705, (95% CI: 0.599, 6.811), *P* = 0.019] and week four [*β* = 4.939, (95% CI: 0.476, 9.401), *P* = 0.030].
Amini et al. [49]	Middle East, Iran	Muslim	Adult Muslim gastrointestinal cancer patients admitted to an inpatient ward at a public hospital in Northwestern Iran	Randomized trial (3 days/until discharge)	Usually two 30–45-minute researcher-led sessions per day for 3 days, plus one ~50-minute session with a clergyman on day 3, that included relationship building (e.g., active listening), spiritual needs and rituals assessment, and spiritual distress assessment. Followed by development/implementation of spiritual care plan, including supportive presence, spiritual ceremonies (e.g., prayer), exploring meaning, bibliotherapy (e.g., reading spiritual books), helping to connect with God, aiding in meeting with loved ones, assisting resolving concerns about suffering, raising hope, recognizing the value of life, finding meaning, building sense of control, building coping skills; Control: Treatment as usual	Templer’s Death Anxiety Scale (T-DAS)	Pre-intervention, death anxiety (T-DAS) was similar between intervention and usual care control groups [8.14 ± 1.54 vs. 8.03 ± 0.85, *P* = 0.429, respectively]. After the intervention, T-DAS was less than the usual care group [7.86 ± 1.22 vs. 8.18 ± 0.79, *P* = 0.029, respectively]. This was not considered a clinically meaningful change, however.
Mahdavi et al. [53]	Middle East, Iran	Muslim	Caregivers of Alzheimer’s dementia pts registered with the Iran Alzheimer’s Association	Randomized controlled trial	Spiritual group therapy for caregivers, over the course of 5 weeks; Controls: (1) attention control—group sessions without spiritual content; (2) no-intervention control	Caregiver strain (Caregiver Strain Index, CSI)	The spiritual group therapy mean of the post-test caregiver strain score [32.43 ± 2.73] was significantly lower than pretest [37.16 ± 1.26] [*P* < 0.001]. The mean post-test score of caregiver strain was significantly lower in the intervention group compared to the two other groups [*P* < 0.001].
Kang et al. [50]	Mixed, Canada, Korea, Hong Kong, Iran, US RCTs and other		Advanced cancer pts	Meta-analysis	Meaning-centered interventions, including: meaning-centered psychotherapy, logotherapy, or meaning-making interventions	Effect sizes by well-being outcomes, categorized as: meaning in life, spiritual well-being, QoL, anxiety, physical symptoms	10 studies included in the meta-analysis (6 randomized, 4 non-randomized) with interventions being meaning-centered psychotherapy, logotherapy, or meaning-making interventions. Studies categorized by outcomes assessed: meaning-in-life (6 studies) [effect size = −0.96 (95% CI: −1.28, −0.64), *P* < 0.001]; spiritual well-being (3 studies) [effect size = −0.37 (95% CI: −0.61, −0.13), *P* = 0.002]; QoL (6 studies) [effect size = −0.48 (95% CI: −0.67, −0.28), *P* < 0.001]; anxiety (3 studies) [effect size = −0.28, (95% CI: −0.52, −0.04), *P* = 0.02]; physical symptoms (3 studies) [effect size = −0.31 (95% CI: −0.56, −0.05), *P* < 0.001].
Lowther et al. [39]	Sub-Saharan Africa, Kenya		Adult HIV pts, on ART for at least 1 month, with a pain or symptom score of 3-5 (from a possible range of 0-best to 5-worst) on the African Palliative Care Association Palliative Outcome Scale (APOS), being seen at an HIV clinic in Mombasa	Randomized controlled trial	Intervention: six contacts over 5 months (baseline, 2 wk, 4 wk, then 3 monthly) with a palliative-care-trained HIV nurse who provided assessment and a care plan addressing physical, psychological, social and spiritual problems; complex pts referred to specialist palliative care. Control: usual HIV clinic care (monthly appointments). 1:1 randomization, *n* = 60 per arm.	Care received (Client Services Receipt Inventory, CSRI); Qualitative exit interviews to further assess pt perceptions of the intervention; Mental health (MOS-HIV)	A RCT of a nurse-led palliative care intervention for pts with HIV found in assessments of 22 components of the palliative care intervention (medication and psychosocial), discussion about spiritual worries was higher in the intervention arm vs. usual care [95% vs. 57%, *P* < 0.001], emotional support from staff [100% vs. 80%, *P* < 0.001], use of weak opioids [60% vs. 18%, *P* < 0.001], discussion about the future [67% vs. 22%, *P* < 0.01], constipation medication [27% vs. 12%, *P* = 0.04], support for the family in planning for the future [65% vs. 22%, *P* < 0.001]; visits from spiritual leaders did not differ [68% vs. 63%, *P* = 0.56]. Clinically significant mental-health improvement (MOS-HIV MHSS) was numerically higher in the intervention arm [57% vs. 52%; not formally tested].
Weru et al. [51]	Sub-Saharan Africa, Kenya		Adult advanced cancer (stage 3 and 4) patients receiving care at clinical settings of the Aga Khan University Hospital Nairobi	Randomized trial (6 weeks)	Dignity therapy; Control: Treatment as usual	Quality of Life (Edmonton Symptom Assessment Scale, ESAS)	There was no significant difference between ESAS Quality of life in the intervention versus the control group.

### Intervention characteristics

Interventions varied in intensity (1–12 sessions) and delivery mode (individual, group, or dyadic), but all focused on spiritual well-being, meaning, and existential coping. The Iranian SC program delivered personalized, face-to-face sessions over three days, incorporating prayer, bibliotherapy, and hope-focused dialogue [49]. The Meaning-Centered Intervention meta-analysis synthesized 10 trials, 6 of them randomized (623 patients), and showed benefits from 2 to 12 sessions of logotherapy-based meaning enhancement [50]. Nurse-led palliative care in Kenya embedded holistic assessment of physical, psychological, social, and spiritual needs into HIV care, extending consultations from 8 to ~45 minutes [39]. Spiritual group therapy for Iranian Alzheimer’s caregivers involved 5 weekly 45 to 60-minute sessions that combined Qur’an recitation with discussion of divine themes [53]. Family Participatory Dignity Therapy [54] and Family-Oriented Dignity Therapy [52] in China employed two to three structured interviews to co-create personalized legacy documents and enhance communication. Individual Dignity Therapy in Kenya involved one 30 to 60-minute session, utilizing 10 life-reflection questions to generate a written “legacy” document [51]. Collectively, these SC approaches emphasize spiritual meaning, relational connection, and culturally grounded practices as core components.

### Outcomes and findings

A meta-analysis showed that meaning-centered interventions significantly improved meaning in life, with smaller but substantial gains in QoL, spiritual well-being, and anxiety [50]. A brief 3-day Iranian SC program reduced death anxiety among gastrointestinal cancer patients, although not clinically meaningful [49]. In Kenya, a nurse-led palliative care intervention for people living with HIV improved mental health and the quality of physician communication, leading to increased discussions of spiritual concerns (95% vs. 57%; *P* < 0.001) and planning ahead for family (65% vs. 22%; *P* < 0.001) [39]. Spiritual group therapy reduced strain among Iranian caregivers of Alzheimer's disease (AD) patients (*P* < 0.001) [53]. Family Participatory Dignity Therapy improved patients’ hope, spiritual well-being, and family cohesion, while reducing caregiver anxiety and depression [54]. Similarly, Family-Oriented Dignity Therapy for lung cancer reduced existential distress (*β* = −1.37; *P* = 0.003), decreased depression (*β* = −3.43; *P* < 0.001), and improved spiritual well-being at one and four weeks [52]. In contrast, a Kenyan RCT of single-session Dignity Therapy found no significant QoL benefit, though anxiety showed a medium effect size improvement (*d* ≈ 0.5) [51]. Spiritually informed interventions, particularly meaning-centered and dignity-based approaches, enhance meaning, hope, and spiritual well-being [49–54]. Family-integrated formats achieved the most sustained benefits while brief or single-session models produced modest gains.

## Religious Affiliation and Spirituality in LMICs

In Muslim-majority settings, prayer and a relationship with God were the most common sources of hope and coping. Greater spiritual well-being was associated with better mood and greater hope [11, 31, 35]. An Iranian ICU study found an association between higher spirituality and lower depression, but greater post-traumatic stress, emphasizing the psychological complexity of faith under serious illness [32]. Although SC was normatively supported, clinician-led assessments in these settings were rare, largely due to limited training [19, 20, 48].

In Christian-majority cohorts, including Latin America and South Africa, SC was associated with less pain, more home deaths (vs. hospitalized), and better psychosocial outcomes [26]. Across Brazilian and multinational samples, positive religious coping correlated with higher QoL and lower distress, whereas religious struggle predicted anxiety and impaired well-being [34].

In Buddhist-majority Thailand, spirituality centered on existential preparation for death and the cultivation of meaning. Higher spiritual well-being was associated with greater perceived effectiveness of palliative care [17], and patients most valued “preparing for death” and “finding life purpose” [29]. Finally, in non-religious or mixed settings (e.g., China), spiritual well-being was typically lower. Nevertheless, individuals reported benefits from interventions that fostered peace, connection, and meaning rather than explicit religiosity [18, 36, 54]. Overall, spirituality and religion were salient, measurable, and generally associated with improved mental, social, and existential outcomes across traditions, with the strongest associations observed for meaning-related and affective dimensions of well-being.

## SC Assessments Rely on Methods Developed in HIC Settings

**Figure 2:**
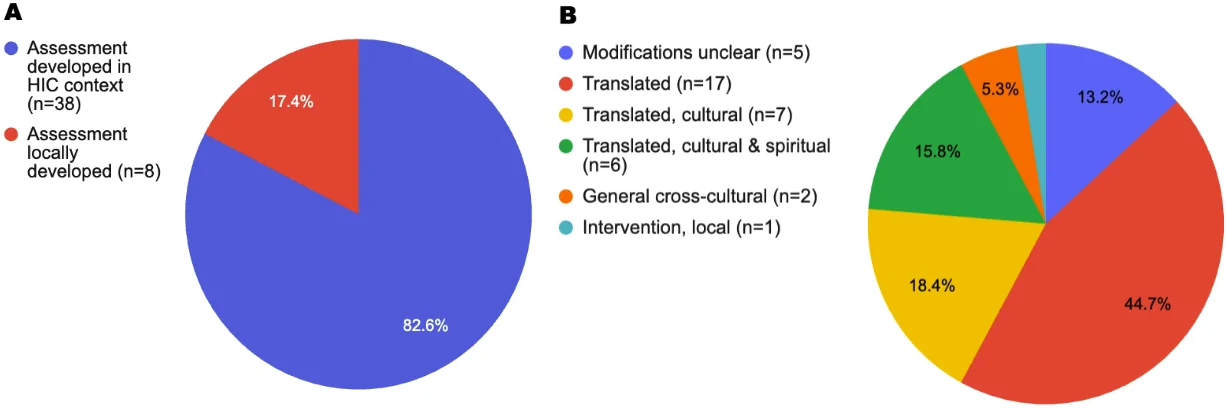
SC assessments.

Of the 46 studies, 38 (83%) used assessments that were originally developed in HIC settings, most of which were translated, validated, or culturally adapted for local use. Across these, forward–backward translation, expert review, pilot testing, and reliability checks were standard. Several studies reported cognitive interviewing (e.g., Satija et al. [46] for the Hindi FACIT-Sp). A smaller subset employed locally developed measures (e.g., the Iranian Palliative-Care Needs Scale, the Thai Spiritual Needs Scale for terminal cancer, and the Nigerian Spiritual-Issues Checklist) [29, 38, 45]. Among the HIC tools, 13 studies explicitly reported additional modifications, including tradition-specific terms or restructuring items to improve conceptual fit (Figure 2). For example, in Ghana, the SNAP was administered in Twi with contextualized wording (e.g., “visits from clergy/imam of your own faith community”) [12]. SNAP comprises 23 items grouped into psychosocial, spiritual, and religious domains and was designed to be multi-faith (e.g., item examples reference the Torah, Qur’an, Bible, Analects, and Tibetan Book of the Dead). Other examples of adaptation include the SWBS, edited for the Thai Buddhist context [17]; the Arabic Beliefs and Values Scale, revised with faith-specific wording and item deletion to suit Jordanian culture [21]; and the Brazilian Brief RCOPE, in which the “demonic reappraisal” item did not load cleanly and was handled separately due to cultural semantics [34]. Several teams employed measures developed to be religion-agnostic, such as the EORTC QLQ-SWB32 [18], or followed standardized translation protocols across countries [24].

In contrast, few studies explicitly incorporated context-specific practices within their research processes (Figure 2). Although excluded from our primary analysis due to quality concerns, Sankhe et al. [55] drew on Hindu practices (e.g., austerities/mercy toward animals), and Han et al. [56] applied combined Naikan and Morita therapies in China (Naikan derives from Jodo Shin Buddhist practice but was secularized by its founder for general use) [55, 56].

Finally, a South African study combined Brief RCOPE items with African Palliative Care Association Palliative Outcome Scale (APCA POS) existential items (“felt at peace,” “life worthwhile”) and directly asked about traditional healers/medicine, but also noted gaps in assessing ancestral beliefs, suggesting these require bespoke tools [26]. Similarly, cross-regional work faced adaptation constraints (e.g., resource-limited translations), highlighting that even well-validated instruments may miss salient, culture-bound constructs [47].

## Discussion

This narrative synthesis of 46 empirical studies with low or moderate risk of bias constitutes, to our knowledge, the most rigorous and comprehensive summary to date of SC in serious illness across LMICs. Across diverse regions, illnesses, and religious traditions, three findings stand out. First, spiritual concerns are pervasive and clinically salient, with spiritual well-being and positive religious coping consistently associated with better QoL, psychological adjustment, and family outcomes. Second, spiritual needs remain substantially unmet despite strong endorsement of SC by both patients and clinicians. Third, SC provision in LMICs is shaped by distinctive patterns in delivery systems, religious landscapes, and measurement practices. Since most of the evidence is cross-sectional and several regions are represented by only a few studies, the associations summarized here describe consistent patterns rather than established causal effects.

In many LMICs, family, religious leaders, and community networks, rather than clinicians, provide SC, reflecting cultural strengths but potentially disadvantaging patients lacking such networks [12, 16, 19]. This pattern invites reflection on how SC is conceptualized. In much of the HIC empirical literature, SC is framed as a professional service located within the health system, delivered by trained clinicians and chaplains. In many LMIC settings, SC instead functions as an existing social practice embedded in family life and faith communities, one that precedes and often operates independently of formal services. An implication for health systems is not merely to introduce SC as part of care delivery where it is absent, but to simultaneously recognize, support, and coordinate with care already occurring (e.g., through community structures), while ensuring that patients without strong family or faith networks are not overlooked. We suggest that neither model is inherently superior. Each carries distinct risks. For example, professionalized SC is often overlooked by physicians, undervalued by hospital systems, and may feel institutional or inaccessible to patients, all of which make trusted community structures more critical. Community-dependent SC, on the other hand, may leave isolated patients unserved and its quality unexamined. Recognizing SC provision as varying along this professional–communal continuum, shaped by health system development and the local religious community, may be more useful than treating HIC delivery models as the default endpoint toward which LMIC systems should converge. Additionally, clinicians’ beliefs and training influenced SC provision, with those in lower-HDI settings offering SC more often, although this finding comes from a study in a single region [13, 14]. Notably, as services shift toward allopathic, medicalized structures, communal structures that provide SC may weaken or be overlooked [13, 14]. This pattern follows HICs, which have moved away from historical affiliations with religious bodies and are now seeking to reintegrate SC [57]. Rather than supplanting traditional SC sources, structured systems could preserve their relational ethos while ensuring equitable reach [58]. Embedding some training or exposure to SC in medical and nursing education would also enable clinicians to provide SC in routine practice. Beyond clinician training, culturally responsive models of care could formally incorporate the actors who already deliver most SC in these settings. Family-integrated intervention formats, which achieved the most sustained benefits in our synthesis [52, 54], offer one potential avenue. Others may involve structured partnerships in which faith leaders receive basic serious-illness communication training, and lay or community health workers conduct spiritual screening with referral pathways to clinicians or chaplains where available.

### Regional and religious tradition patterns

Spirituality emerged as a universal yet locally inflected dimension of serious-illness care. Despite regional differences, higher spiritual well-being and positive religious coping were consistently associated with better QoL and lower distress. In contrast, religious/spiritual struggle was associated with poorer mental health and QoL. This pattern mirrors findings from Western and other HIC settings, suggesting that aspects of SC in serious illness may be generalizable rather than LMIC-specific. A meta-analysis of 78 samples and 14,277 cancer patients, 56% of them conducted in North America, similarly found religion and spirituality associated with better social health (Fisher *z* = 0.20, *P* < 0.001), indicating that this pattern is not confined to LMIC settings [59].

However, several features characterize LMICs, including the primacy of family and faith communities as SC providers, the inverse association between national HDI and clinical SC provision, and the overlap between spiritual concerns, poverty, and constrained access to services. Collectively, these results support the need for practical SC partnerships with families, faith leaders, and the community to attend to person-centered care—rather than secular biomedical models that may overlook patients’ spiritual and social dimensions [12, 19]. However, because most regions are represented by a small number of studies, often from single institutions, we cannot determine which patterns are truly region-specific and which reflect idiosyncrasies of particular study populations. Our regional observations should therefore be treated as hypotheses for larger comparative work, not as definitive claims of cultural uniqueness.

### Interventions

Although intervention studies remain few, they show that SC can be delivered feasibly and with measurable benefit across settings. Nurse-led, family-integrated, and dignity-based interventions improved communication, meaning, and emotional well-being for patients and caregivers [39, 52–54]. Brief or single sessions produced weaker effects, highlighting the value of sustained engagement [49–51]. Meaning-centered interventions demonstrated consistent improvements in well-being and reduced distress [50]. Collectively, these findings position SC as a core, relational component of person-centered serious-illness care in LMICs.

### Methods and measurement

Most measures were developed in HIC settings but have been successfully translated and validated for LMIC contexts. However, heavy reliance on imported tools may risk neglecting local spiritual elements. Locally developed instruments from Thailand, Iran, and Nigeria illustrate that context-specific measures are feasible and can enrich the field by revealing culturally distinctive pathways to spiritual well-being [29, 38, 45]. The APCA African Palliative Outcome Scale, used in three of the African studies reviewed here, extends this to a regional scale. The African Palliative Care Association developed it through item generation with African patients and families, then validated it across multiple sub-Saharan African sites. Multicenter factor analysis in the region established three subscales, including an existential and spiritual well-being domain. As Figure 2 illustrates, most included studies relied on translated HIC instruments, a smaller number adapted these tools to local traditions, and only a few drew on locally developed measures or context-specific practices. Rather than viewing HIC and LMIC approaches as competing, we suggest a “both/and” framework that retains validated global measures for cross-cultural comparison while integrating locally grounded tools to capture lived spiritual experiences. This dual approach allows researchers to preserve comparability without sacrificing cultural fidelity.

### Exploring the higher-level outcomes of spiritual care—meaning, hope, and purpose

Evidence across settings indicates that SC contributes to psychological and existential adjustment by helping patients interpret suffering, sustain hope, and find coherence amid illness. These associations echo Park’s integrative model in which adjustment arises from reconciling beliefs and experiences through meaning reconstruction [60]. Empirically, studies show that spiritual well-being and perceived meaning are closely linked with QoL, lower distress, and family cohesion. In contrast, religious or spiritual coping moderates the effects of illness-related adversity [61–64]. Hope co-occurs with meaning, and those who sustain hope during illness report better psychological outcomes near the end of life [65, 66]. Dignity- and meaning-centered psychotherapies improve well-being across high- and low-income settings [52, 54, 67–69].

## Limitations

This review has several limitations. As a secondary analysis of a parent systematic review, it inherits previously identified limitations, including reliance on the original study selection, data extraction, and risk-of-bias judgments. The LMIC evidence base remains thin and uneven, with many regions and religious traditions represented by single, relatively small, hospital-based samples. Most studies were cross-sectional surveys conducted in specialist or tertiary-care settings, using self-report instruments. This limits causal inference, constrains generalizability, and under-represents patients managed in primary care, community, or home-based services, as well as family caregivers and people with non-cancer serious illness. Sex-disaggregated analyses were not conducted in this review because the parent systematic review did not extract sex-stratified data in a form that would permit systematic synthesis across the included studies. Heterogeneity in populations, measures, and outcomes precluded meta-analysis, so our narrative synthesis is vulnerable to selective reporting and publication bias.

## Conclusions

Despite these limitations, coherence with Balboni et al. [1] strengthens the conclusion that SC is a fundamental dimension of serious-illness care. The LMIC literature reviewed here, though still developing and unevenly distributed across regions, demonstrates methodological rigor and practical feasibility. Together with the HIC literature, our findings suggest that the association among spiritual well-being, religious coping, QoL, and psychological distress is consistent across settings, although largely based on cross-sectional evidence. However, patterns such as the dominance of family and faith networks in providing SC, the inverse gradient in HDI-SC provision, and the link between spiritual concerns and structural disadvantage may characterize LMIC contexts. We recommend building on this foundation by developing culturally grounded, scalable SC models co-designed with clinicians, patients, families, and faith/community partners. Combining globally validated instruments with locally relevant measures, and extending research beyond cross-sectional designs to include longitudinal and interventional studies, will strengthen the evidence base. In particular, multi-country consortia that intentionally include sites from multiple LMIC regions and HIC comparators may distinguish context-specific phenomena from globally shared SC features. Given the small number of studies per region in this review, our synthesis provides a map of promising directions rather than definitive statements about cultural uniqueness. Embedding indicators of spiritual well-being within serious-illness and palliative-care quality frameworks and incorporating SC competencies into medical and nursing education may help align health systems with what patients and caregivers consistently value—meaning, connection, and purpose. As the evidence base advances, consolidating it through culturally responsive implementation and sustained evaluation will help make spiritual care a core element of person-centered healthcare in LMICs.

## Data Availability

All authors had access to the data and contributed to the writing of the manuscript. No new data were generated for this study. The narrative synthesis draws on previously published literature. The full list of included studies, extraction tables, risk-of-bias assessments, and supporting data for the analyses is provided in Supplementary Material S1–S4.

## Author Access to the Data

All authors had access to the data and contributed to the writing of the manuscript (see below).

## Additional File

The additional file for this article can be found as follow:
