# Supplementary material for: Spiritual Care in Serious Illness: A Narrative Review of Low- and Middle-Income Country Evidence
Source: Ann Glob Health. 2026 Sep 18;92(1):98. doi: 10.5334/aogh.5384 (PMC13588206; doi:10.5334/aogh.5384)
Supplement: Supplementary file 1 [file agh-92-1-5384-s1.pdf]

Supplementary material

Supplementary S1: **Studies excluded due to serious/critical bias concerns**

1. Bhatnagar S, Gielen J, Satija A, Singh SP, Noble S, Chaturvedi SK. Signs of Spiritual Distress and its Implications for Practice in Indian Palliative Care. Indian J Palliat Care. 2017 Jul-Sep;23 (3):306-311. doi: 10.4103/IJPC. IJPC_24_17. PMID: 28827935; PMCID: PMC5545957."
2. Camargos MG, Paiva CE, Barroso EM, Carneseca EC, Paiva BSR. Understanding the Differences Between Oncology pts and Oncology Health Professionals Concerning Spirituality/Religiosity: A Cross-Sectional Study. Medicine (Baltimore). 2015 Nov;94(47):e2145. doi: 10.1097/MD. 0000000000002145. PMID: 26632743; PMCID: PMC5059012. [for high risk of bias in the role of spirituality, but not Spiritual Care)
3. Cheawchanwattana A, Kanjanabuch T, Puapatanakul P, Narenpitak S, Halue G, Tungsanga K, Tatiyanupanwong S, Lorvinitnun P, Sritippayawan S, Chieochanthanakij R, Tungsanga S, Thamcharoen N, Pongpirul K, Shen JI, Johnson DW, Davies SJ, Finkelstein FO, Perl J, Robinson B; Thailand PDOPPS Steering Groups. Spiritual well-being and its relationship with patient characteristics and other patient- reported outcomes in peritoneal dialysis patients: Findings from the PDOPPS. Nephrology (Carlton). 2022 Mar 3. doi: 10.1111/nep.14034.Epub ahead of print. PMID: 35238113."
4. Deng D, Deng Q, Liu X, Xie CH, Wu X. Expectation in Life Review: A Term of Spiritual Needs Easily Understood by Chinese Hospice pts. Am J Hosp Palliat Care. 2015 Nov;32(7):725-31. doi: 10.1177/1049909114539727. Epub 2014 Jun 24. PMID: 24963084.
5. Duman M, Durgun Ozan Y, Doğan Yüksekol Ö. Relationship between the religious attitudes of women with gynecologic cancer and mental adjustment to cancer. Palliat Support Care. 2021 Feb;19(1):62-68. doi: 10.1017/S1478951520000619. PMID: 32744209."
6. Garg R, Chauhan V, Sabreen B. Coping Styles and Life Satisfaction in Palliative Care. Indian J Palliat Care. 2018 Oct-Dec;24(4):491-495. doi: 10.4103/IJPC.IJPC_63_18. PMID: 30410263; PMCID: PMC6199830.
7. Han XB, Fang YQ, Liu SX, Tan Y, Hou JJ, Zhao LJ, Li F. Efficacy of combined naikan and morita therapies on psychological distress and posttraumatic growth in Chinese patients with advanced cancer: A randomized controlled trial. Medicine (Baltimore). 2021 Jul 30; 100(30):e26701. doi: 10.1097/MD.0000000000026701. PMID: 34397698; PMCID: PMC8322545."
8. Hatefi M, Vaisi-Raygani A, Borji M, Tarjoman A. Investigating the Relationship between Religious Beliefs with Care Burden, Stress, Anxiety, and Depression in Caregivers of Patients with Spinal Cord Injuries. J Relig Health. 2020 Aug;59(4):1754-1765. doi: 10.100/s10943-019-00853-3. PMID: 31187306."
9. Kavak Budak F, Özdemir A, Gültekin A, Ayhan MO, Kavak M. The Effect of Religious Belief on Depression and Hopelessness in Advanced Cancer Patients. J Relig Health. 2021 Aug;60(4):2745-2755. doi: 10.1007/s10943-020-01120-6. Epub 2021. Jan 3. PMID: 33389479."
10. Lazenby M, Khatib J. Associations among pt characteristics, health- related quality of life, and spiritual well-being among Arab Muslim cancer pts. J Palliat Med. 2012 Dec; 15(12):1321-4. doi: 10.1089/jpm. 2012.0208. Epub 2012 Sep 28. PMID: 23020068.
11. Musarezaie A, Naji-Esfahani H, Momeni-Ghale Ghasemi T, Karimian J, Ebrahimi A. Investigation of the SWB and its relation with demographic parameters in pts with breast cancer referred to an oncology hospital affiliated to the Isfahan university of medical sciences. J Educ Health Promot. 2013 Jul 31;2:42. doi: 10.4103/2277- 9531.115844. PMID: 24251278; PMCID: PMC3826021.
12. Paiva CE, Paiva BS, Yennurajalingam S, Hui D. The impact of religiosity and individual prayer activities on advanced cancer pts' health: is there any difference in function of

whether or not receiving palliative anti-neoplastic therapy? J Relig Health. 2014 Dec;53(6):1717-27. doi: 10.1007/s10943-013-9770-6. PMID: 23990104.

13. Sankhe A, Dalal K, Agarwal V, SarveP. Spiritual Care Therapy on Quality of Life in Cancer pts and Their cgs: A Prospective Non-randomized Single- Cohort Study. J Relig Health. 2017. Apr;56(2):725-731. DOI:10.1007 /s10943-016-0324-6. PMID: 27853982 PMCID:PMC5320005.""Sastra, L.; Bussing, A.; Chen, C. H.;
14. Yen, M. F.; Lin, E. C. L.. Spiritual Needs and Influencing Factors of Indonesian Muslims With Cancer During Hospitalization. Journal of Transcultural Nursing May 2021;32 (3):212-220. DOI: 10.1177 /1043659620908926."
15. Wang Z, Wang Z, Wang Y, Xiao L, Zhao H, Li X, Zhang S, Pang X. Spiritual care needs and their associated influencing factors among elderly patients with moderate-to-severe chronic heart failure in China: A cross-sectional study. Palliat Support Care. 2022 Apr;20(2):264-274. doi: 10.1017 /S1478951521001279. PMID: 35574915."

Supplementary Table S2: All included studies (N=46)

Author, Year	Population	N	Design	R/S variables	Summary of results	Outcome variables	Bias rating	Bias description	Measurement
Chaiviboontham, 2015	**East Asia, Thailand Adult advanced cancer pts seen receiving palliative care three hospitals in Bangkok and suburban Thailand**	240	Cross-sectional survey study	Spiritual Well-Being Scale (SWBS) Personal Information Questionnaire (PIQ)	41.7% perceived palliative care interventions e.g., pharmacologic and non-pharmacologic) to be effective. In MVA, spiritual well being (SWBS) was associated with greater pt-perceived efficacy of palliative care [b=0.057, SE=0.012, p<0.001].	Effectiveness of palliative care (Palliative Care Assessment Form, PCAF)	Low	Cross-sectional study using validated scales; sample size=240 from three medical institutions; RR=75%; MVA control for potential confounds to characterize cross-sectional associations; generalizability considerations due to population studied (adult advanced cancer pts receiving palliative care in Thailand).	**Method: Western, modified, translation, cultural context, spiritually specific**
Chen et al. 2021	**East Asia, China Adult gynecological cancer patients recruited from a single university hospital in western China**	705	Cross-sectional survey study	Spiritual well-being (European Organisation for Research and Treatment of Cancer spiritual well-being, EORTC QLQ-SWB32)	In MVA, global health [β=0.337, p<0.001] and depression [β=-0.144, p<0.001] were significantly associated with Global-SWB.	Quality of life (European Organisation for Research and Treatment of Cancer quality of life instrument, EORTC QLQ- C30) Depression (Hospital Anxiety and Depression Scale, HADS)	Moderate	Cross-sectional study using validated scales; sample size=705 gynecological cancer patients from a single university hospital in China; RR not reported; MVA examined associations of spiritual well- being with global health and depression; generalizability considerations due to population studied (adult female gynecological patients from a single university hospital in China).	**Method: Western, Cross-culturally developed**
Chui et al. 2014	**East Asia, Malaysia Adult female breast cancer pts recruited from Hospital Kuala Lumpur (HKL) and the University of Malaya Medical Centre (UMMC)**	546	Cross-sectional survey study	Complimentary and alternative medicine (CAM) questionnaire, including massage, exercise, prayer for health (PFH), supplements, herbal remedies, and indigenous medicine	70.7% report using any form of CAM. The largest category of CAM-use was prayer for health [56.8% of full sample, 80.3% of CAM users], and 95.9% find prayer helpful.	Descriptive study of CAM use	Low	Descriptive study using valid scales; sample size=546 from two medical institutions; RR=91.6%; generalizability considerations due to population studied (women with breast cancer in Malaysia).	**Method: Locally Developed**

Effendy et al. 2015	**East Asia, Indonesia Adult, hospitalized cancer (any stage) pts in 3 general hospitals in Indonesia**	150	Cross-sectional survey study	Assessment of pt palliative care needs (Problems and Needs of Palliative Care scale short version, PNPC-sv)	82.4% of pts physical symptoms; 74.8% financial issues; 58.8% psychological issues; 51.3% ADLs issues; 43.7% spiritual issues; autonomy 39.5%; 27.7% social issues. 88.4% indicated spiritual issues were addressed by family [80.7%], nurses [63.4%], MDs [38.4%].	Description of symptoms and spiritual issues	Low	Descriptive study using validated scales; sample size=150 from 3 medical institutions; RR=79%; generalizability considerations due to population studied (adult cancer pts, any stage, cared for at 3 hospitals in Indonesia).	**Method: Western, modified, translation and cultural context**
Kwok et al. 2021	**East Asia, Hong Kong, Taiwan, Thailand Adult Parkinson's disease patients recruited from neurological clinics in China (Hong Kong), Taiwan, and Thailand**	186	Cross-sectional survey study	Unmet supportive care needs, including physical symptoms; emotional, psychosocial, and spiritual needs; and provision of information and practical concerns (Palliative care Outcome Scale, POS, the Chinese version for HK and Taiwan and a Thai version for Thailand)	55.4% reported unmet spiritual needs (POS). Degree of spiritual need varied by country: Hong Kong 70.3%, Taiwan 64.1%, and Thailand 29.3% [p=0.013]. Other common supportive care needs included: physical symptom needs [62.9%], psychosocial needs [64%]; anxiety needs [52.2%].	Symptom burden (Palliative Care Outcomes Symptom - Parkinson's Disease) Quality of life (7-item EQ-5D)	Moderate	Cross-sectional study using validated scales; sample size=186 Parkinson's disease (PD) patients from three countries (China, Taiwan, and Thailand); RR not reported; generalizability considerations due to the population studied (PD patients from China (Hong Kong), Thailand, and Taiwan).	**Method: Western, modified, translation**
Liu et al. 2022	**East Asia, China Adult cancer inpatients and adult non-cancer chronic illness inpatients recruited from the Chongqing University Three Gorges Hospital**	630	Cross-sectional survey study	Spiritual well-being (Functional Assessment of Chronic Illness Therapy, FACIT-Sp, Chinese version)	97% of patients had no religious affiliation; spiritual well being was moderate among cancer patients versus chronic illness (non-cancer) patients; and cancer patients SWB scores were lower than patients with chronic illnesses [FACIT-Sp: μ(cancer pts)=33.30, σ=10.35; μ(chronic illness pts)=38.61, σ=10.88, p<0.001].	Descriptive assessment of spiritual well-being (SWB), with comparisons of SWB between those with cancer versus chronic diseases, and predictors of SWB	Moderate	Cross-sectional study using a validated scale; sample size=630 cancer and chronic non-cancer illness inpatients from a single hospital in China; RR=97%; generalizability considerations due to populations studied (adult cancer inpatients and chronically ill patients recruited from a single hospital in China).	**Method: Western, modified, translation and cultural context**
Myint et al. 2021	**East Asia, Myanmar Adult cancer patients (all stages) seen at 3 oncology clinics in Mandalay, Myanmar**	141	Cross-sectional survey study	Palliative care needs (Palliative care assessment tool, including psychosocial and spiritual needs)	90.1% of patients desired spiritual support. 73.0% wanted help to find meaning in their cancer experience. 85.8% were willing to receive palliative care services. In MVA, greater spiritual and psychosocial need predicted willingness to receive palliative care services	Willingness to receive palliative care services (structural-functional social support scale, SFSS) Social support (structural-functional social support scale, SFSS)	Low	Cross-sectional study using validated scales; sample size=141 cancer patients from 3 oncology clinics in Mandalay, Myanmar; RR=75%; MVA to assess associations of desiring spiritual support with greater willingness to receive palliative care services; generalizability	**Method: Locally Developed**

					[AOR=3.76, (95% CI: 1.17, 12.05),			considerations due to population studied (adult cancer patients seen at 3 oncology clinical sites in Mandalay, Myanmar).	
Wang et al. 2021	**East Asia, China Adult hematological malignancy patients and their informal family caregiver recruited from Fujian Medical University Union, Fujian, China**	136 (68 pt-cg dyads)	Randomized trial (8 weeks)	Intervention: Family participatory dignity therapy Control: Treatment as usual	For patients, there was a significant improvements in hope [p=0.001], spiritual well-being [p=0.002), and family cohesion [p<0.001] and adaptability [p<0.001] between the intervention and control groups. The difference over time was also significant in family cohesion [p=0.018] and adaptability [p=0.003]. The interaction effects were significant for hope [p=0.034], spiritual well-being [p<0.001], and family cohesion [p<0.001] and adaptability [p<0.001]. For family caregivers, there was a significant difference in anxiety [p=0.037], depression [p=0.001], and family adaptability [p=0.036] between the intervention and control groups. Within groups, a significant difference in family adaptability [p=0.012] was found. Moreover, the interaction effects were significant on anxiety [p=0.001] and family cohesion [p=0.038].	Patient hope (Herth hope index, HHI); Patient spiritual well-being (Functional assessment of chronic illness therapy -spiritual well-being scale, FACIT-Sp); Caregiver anxiety (self-rating anxiety scale, SAS); Caregiver depression (self-rating depression scale, SDS); Family adaptability (Family adaptability and cohesion evaluation scale-II, FACES II, for patients and family caregivers	Moderate	Randomized trial of family dignity intervention versus usual care among patients with hematological malignancies, and their informal caregivers, seen at a single hospital in Fujian, China; Consent Rate=61%; generalizability considerations given the population studied (adult, hematological patients and their informal caregivers cared for at a single hospital setting in Fujian, China).	**Method: Western, modified, translation**
Wisesrith et al. 2021	**East Asia, Thailand Adult terminally ill cancer patients from seven hospitals in northern, northeast, central, and southern regions of Thailand**	322	Cross-sectional survey study	Spiritual needs (Spiritual Needs Scale, developed and validated by the authors)	The overall spiritual needs of terminal ill cancer patients were at the moderate level [μ=18.21, σ=2.56]. The highest mean was found in the 'prepare for death' dimension, followed by the 'have meaning, values, and life purposes' and the 'have opportunity to pursue most important things in life' dimensions respectively and	Descriptive study of spiritual needs in Thai patients	Moderate for description of spiritual needs; Serious for predictors of spiritual needs	Cross-sectional survey study of advanced cancer patients seen at 7 hospitals throughout Thailand; RR not reported; univariable analysis performed to assess predictors of spiritual needs; generalizability considerations due to the population studied (advanced cancer patients	**Method: Locally Developed**

					had different spiritual needs among married persons (versus widowed) [F(3,318)=3.66, p<0.05], with increasing number of family members [F(8,313)=5.07, p<0.05], living with family [F(2,319)=3.91,p<0.05], and having a spiritual anchor (key spiritual supporter) [F(2,319)=4.13, p<0.05].			seen at 7 hospitals throughout Thailand).	
Xiao et al. 2022	**East Asia, China Adult lung cancer patients receiving chemotherapy, and their informal caregivers, at a cancer hospital in Changsha, China**	120	Randomized trial (4 weeks)	Intervention: Family- oriented dignity therapy Control: Attention (education) control	Patients in the intervention group showed significantly greater reduction in existential distress [β=-1.372, (95% CI: -2.269, -0.472), p=0.003] and depression [β=-3.430, (95% CI: -5.032,-1.829), p<0.001] at week one, as well as significantly greater improvement in spiritual well-being at both week one [β=3.705, (95% CI: 0.599, 6.811), p=0.019] and week four [β=4.939, (95% CI: 0.476, 9.401), p=0.030].	Dignity (Patient Dignity Inventory), Depression (Patient Health Questionnaire-9), Spiritual well-being (Functional Assessment of Chronic Illness Therapy - Spiritual Well- being Scale, FACIT-Sp)[AA1]	Moderate	Randomized trial of family-oriented dignity therapy versus attention (education) control among 120 lung cancer patients actively undergoing chemotherapy at a single hospital in China; Consent Rate=77%; generalizability considerations due to the population studied (advanced cancer patients seen at a single hospital in Changsha, China).	**Method: Western, modified, translation**
Yang et al. 2021	**East Asia, China Adult advanced metastatic cancer patients with a life expectancy<6 months and admitted to the hospice ward of Shengjing Hospital of China Medical University**	108	Prospective observational study (2 weeks)	Spiritual well-being (Functional Assessment of Chronic Illness Therapy-Spiritual Well- being)	Patients' existential well-being [b=-0.99, (95% CI:-1.72, -0.26), p=0.008] and meaning dimension [b=-0.87, (95% CI: -1.29, -0.43), p<0.001] significantly decreased after admission to the PC unit, but peace and faith did not change over time.	Anxiety and depression (Hospital Anxiety and Depression Scale) Pain (Numerical Rating Scale)	Moderate	Prospective study examining advanced cancer patients admitted to a hospice ward at a single hospital in China; RR=74%; MVA assessed factors contributing to spiritual well-being over time. Generalizability due to population studied (advanced cancer patients admitted to a single hospice ward in China).	**Method: Western, modified, translation**
Demirbag et al. 2018	**Middle East & North Africa, Turkey Nurses who worked at five hospitals in the Eastern Black Sea Region of Turkey**	1,254	Cross-sectional survey study	Characteristics of spiritual care provision (questionnaire developed for study based on literature review)	91% nurses never ask about pt spiritual needs, 98.1% had never received spiritual care training. Training was a strong predictor of assessing spiritual needs [X^2=19.96, p<0.01].	Spiritual care interventions provided by nurses	Moderate	Cross-sectional study using non- validated scale assessing spiritual care perceptions/practices; sample size=1254 from 5 medical institutions; RR=71%; findings largely descriptive, UVA to	**Method: Locally Developed**

								characterize predictors of spiritual care; generalizability considerations due to population studied (nurses, 100% women, 100% Muslim, from the Eastern Black Sea region of Turkey).	
Sahan 2022	**Middle East & North Africa, Turkey Adult patients with COVID-19 admitted to or seen as outpatients to multiple hospitals in Turkey**	384	Cross-sectional survey study	Spiritual needs (Spiritual Care Requirements Scale)	Spiritual needs (Spiritual Care Requirements Scale score) of the participants was 67.05±26.30 (possible 21-105). The mean VAS for Death Anxiety score of the participants was 8.82±1.26 (possible 0-10).	Death anxiety (The Death Anxiety Scale)	Moderate	Cross-sectional survey study of patients with COVID-19 at multiple sites throughout Turkey; RR not reported; generalizability considerations due to the population studied (adult patients with COVID-19 at multiple inpatient and outpatients settings in Turkey).	**Method: Western, modified, translation**
Camargos et al. 2015	**Latin America, Brazil Adult, advanced cancer pts (n=525), and health care professionals providing direct care to oncology pts (n=525) from multiple clinical settings affiliated with a tertiary care cancer center in Sao Paulo State, Brazil**	1,050	Cross-sectional survey study	Assessment of perceptions of spiritual/religious care; Spiritual well-being (World Health Organization QOL: Spiritual, Religious and Personal Beliefs scale, WHOQOL-SRPB)	94.1% of pts considered it important that health professionals ask them about their spiritual beliefs. Nearly all pts [99.6%] reported that spiritual support is necessary during cancer treatment; 98.3% of health professionals agreed that spiritual and religious support was necessary for oncology pts.	Description of pt and MD perceptions of spiritual/religious care; Global pt QOL (World Health Organization Quality of Life Questionnaire, WHOQOL-Brief)	Low	Cross-sectional study using validated scales; sample size=1050 from multiple clinical settings from a cancer center; RR=97.7% pts, RR=97.4% MDs; MVA control for potential confounds to characterize cross-sectional associations; generalizability considerations due to population studied (adults with advanced cancer and MDs caring for oncology pts in Brazil).	**Method: Western, Cross-culturally developed**
Martinez & Custodio. 2014	**Latin America, Brazil Adult end-stage renal disease on hemodialysis pts from a single medical center in Brazil**	150	Cross-sectional survey study	Spiritual well-being (Spiritual Well-Being Scale, SWBS)	In MVA, spiritual well-being (SWBS) predicted better mental health (GHQ) [adjusted r2=0.0124, f=5.165, p=0.0009].	Mental health (General Health Questionnaire, GHQ)	Moderate	Cross-sectional study using validated scales; sample size=150 from 1 medical institution in Brazil; RR=89.3%; MVA control for multiple potential confounds to characterize cross-sectional associations; generalizability considerations due to population studied (adult hemodialysis pts from a	**Method: Western, modified, translation**

								single medical institution in Brazil).	
Ramirez et al. 2012	**Latin America, Brazil Adult End stage renal disease (ESRD) patients from three outpatient hemodialysis units in Brazil**	170	Cross-sectional survey study	Positive religious coping and religious struggle (Brief RCOPE)	In MVA, religious struggle correlated with both depressive [b=0.31, p<0.0001] and anxiety [b=0.36, p<0.0001] symptoms. In MVA, positive religious coping was associated with better overall QOL [b=0.17, p=0.02], while religious struggle was associated with worse overall QOL [b=-0.26, p<0.001].	Depression and anxiety (Hospital Anxiety and Depression Scale, HADS); Quality of life (the World Health Organization QoL instrument, abbreviated version, WHOQOL-Brief)	Low	Cross-sectional survey study using validated scales; sample size=170 from 3 hemodialysis clinics in Brazil, RR=89.5%; MVA adjusted for multiple potential confounders; generalizability considerations due to population studied (adult with end stage renal disease in three sites in Brazil).	**Method: Western, modified, translation**
Valentino et al. 2022	**Latin America, Brazil Adult patients (and adult caregivers) with advanced incurable cancer, undergoing systemic palliative treatment and/or individualized palliative care, life expectancy >3 an ≤12 months, performance status ≤3, full cognitive capacity and coherent communication skills recruited from the Barretos Cancer Hospital in Brazil.**	190 pts, 190 cgs	Cross sectional survey study	Spirituality (1 of 6 End of Life factors of importance rated by patients and caregivers)	Of 6 factors most important at the end-of-life, patients and caregivers reported that presence of loved ones [42.3% pts, vs. 44.2% cgs] was the factor of greatest importance, followed by spirituality [38.5% pts vs. 27.9% cgs] and the place-of-death [14.0% pts vs. 18.4% cgs].	Preferred place of death outcome (questionnaire developed/validated by the research team). End of life factors of importance (scale) Depression and anxiety (Hospital Anxiety and Depression Scale, HADS)	Moderate	Cross-sectional study using validated scales; sample size=190 advanced cancer patients, and their informal caregivers, from a single cancer hospital in Brazil; RR=89%; generalizability considerations due to populations studied (advanced cancer patients, and their informal caregivers, cared for at a single cancer hospital in Brazil).	**Method: Western, modified**
Vigna et al. 2020	**Latin America, Brazil Adult informal caregivers of**	178	Cross-sectional survey study	Spirituality, religiousness and personal beliefs (WHO Quality of life - Spirituality, religiousness and personal	Higher spirituality as measured by the SRPB score was associated with lower Zarit scores [r=-0.45,	Psychological morbidity (Self-Reporting Questionnaire, SRQ-	Moderate	Cross-sectional survey study of caregivers of seriously ill patients receiving palliative care at a	**Method: Western, modified, translation**

	**seriously ill patients receiving palliative care at a the Hospital da Luz, in São Paulo, Brazil**			beliefs, WHOQOL-SRPB, scale)	p=0.001] and also with lower SQR-20 score [r=-0.37, p<0.001]; In MVA, spirituality predicted for less burden [b=-0.53, p<0.001].	20) Caregiver burden (Zarit Burden Interview, ZBI) Quality of life (WHOQOL)		single hospital in Brazil; RR=96%; MVA employed to assess relationship of psychological well-being measure with spiritual well being, adjusting for potential confounding factors.	
Afrooz et al. 2014	**Middle East, Iran Adult, inpatient and outpatient cancer pts recruited from Ardebil University of Medical Sciences, Ardebil, Iran**	200	Cross-sectional survey study	Hope Health Index Sources of Hope	Of 14 sources of hope assessed, top 5 for pts: God 100%, prophets/imams 91%, child 67.5%, MDs 63.5%, medicines and treatments available for the disease 62%. Of 20 hope-inspiring strategies assessed, top 5: 97% relationship with God, 87% blessing, 84.5% controlling symptoms of disease, 82% praying, 66% appropriate manner of physician.	Description of sources of hope	Low	Descriptive study using validated scales; sample size=200 from 1 medical institution; RR=87%; generalizability considerations due to population studied (adult cancer pts seen as inpatient or outpatients in Iran).	**Method: Western, modified, translation and cultural context**
Amini et al. 2020	**Middle East, Iran Adult Muslim gastrointestinal cancer patients admitted to an inpatient ward at a public hospital in Northwestern Iran**	162	Randomized trial (3 days and until discharge)	Intervention: Two 40- 50min sessions per day for 3 days that included relationship building (e. g., active listening), spiritual needs and rituals assessment, and spiritual distress assessment. Followed by development/implement ation of spiritual care plan, including supportive presence, spiritual ceremonies (e. g., prayer), exploring meaning, bibliotherapy (e.g., reading spiritual books), helping to connect with God, aiding in meeting with loved ones, assisting resolving concerns about suffering, raising hope, recognizing the value of life, finding meaning, building sense of control, building coping skills Control: Treatment as usual	Pre-intervention, death anxiety (T-DAS) was similar between intervention and usual care control groups [8.14±1.54 vs. 8.03±0.85, p=0.429, respectively]. After the intervention T-DAS was less than the usual care group [7.86±1.22 vs. 8.18 ±0.79, p=0.029, respectively]. This was not thought to be a clinically meaningful change, however.	Templer's Death Anxiety Scale (T-DAS)	Moderate	Randomized controlled trial using validated scales (usual care as control); sample size=162 gastrointestinal cancer pts at one inpatient setting in Northeastern Iran; RR=87.1%; spiritual care content variable per patient, with only description of possible content; generalizability considerations given population studied (gastrointestinal cancer pts at a single inpatient site in Iran).	**Method: Western, modified. Intervention: Locally developed**

Bar-Sela et al. 2019 (B)	Middle East (14 countries) Cyprus, Egypt, Iran, Iraq, Israel, Jordan, Lebanon, Pakistan, Palestine, Saudi Arabia, Sudan, Oman, Turkey, United Arab Emirates Physicians and nurses treating pts with advanced cancer	770	Cross-sectional survey study	Spiritual Care provision and receipt questionnaire; Spiritual care example; Human Development Index (survey developed by United Nations Development programme: composite of life expectancy, education, per capita economic production)	72% Muslim, 13% Jewish, 10% Christian. 90% at least slightly spiritual; 85% at least slightly religious. 82% believe spiritual care (SC) should be provided to advanced cancer pts at least occasionally, but 44% provide SC less often than they think they should. 77% had never received spiritual care training; 77% are interested in receiving training. In MVA of respondents who value spiritual care but don't provide it to pts, predictors included low personal spirituality [p<0.001] and not having received training [p=0.02]. How "developed" (Human development index) a country also negatively predicted spiritual care provision [p<0.001].	Description of perceptions of spiritual care; Predictors of spiritual care provision	Low	Cross-sectional study using validated scales; sample size=770 from multiple medical institutions in 14 countries; RR=79% nurses; 63% MDs; MVA with control for potential confounders to characterize cross-sectional associations; generalizability considerations due to population studied (MDs and nurses treating pts with advanced cancer in 14 Middle Eastern countries).	**Method: Western, unmodified**
Bar-Sela et al. 2019 (A)	**Middle East (14 countries) Cyprus, Egypt, Iran, Iraq, Israel, Jordan, Lebanon, Pakistan, Palestine, Saudi Arabia, Sudan, Oman, Turkey, United Arab Emirates MDs and nurses treating pts with advanced cancer**	770	Cross-sectional survey study	Spiritual care perceptions and receipt/practices (Spiritual Care Provision and Receipt, SCPR); Human Development Index (survey developed by United Nations Development programme: composite of life expectancy, education, per capita economic production)	Respondents were from 14 Middle Eastern countries, 40% MDs/60% nurses; 61% female; 73% Muslim; 25% very high human development index (HDI), 41% high HDI, 29% medium HDI, and 5% low HDI. Of 8 spiritual care types, 42%-81% considered appropriate. Respondents reported providing some form of spiritual care to 47% of their three most recent patients with advanced incurable cancer (physicians 41%, nurses 50%). In MVA, HDI was the strongest factor: staff in high-HDI countries had higher odds of not providing spiritual care than those in medium-HDI countries [AOR=4.21 (95% CI: 2.58, 6.87)], equivalent to greater provision in medium-HDI	Description of frequency and perception of importance of spiritual care provision, impact of spiritual care, and spiritual care training; Assessment of predictors of spiritual care provision	Low	Cross-sectional study using validated scales; sample size=770 from multiple medical institutions in 14 countries; RR=79% nurses; 63% MDs; MVA with control for potential confounders to characterize cross-sectional associations; generalizability considerations due to population studied (MDs and nurses treating pts with advanced cancer in 14 Middle Eastern countries).	**Method: Western, unmodified**

					settings. Other significant predictors were spirituality, intrinsic religion/spirituality, training and being a nurse. Only 22% had received spiritual care training, 77% indicated interest in receiving spiritual care training.				
Bashar et al. 2018	**Middle East, Iran Adult pts who had been admitted to the ICU and received mechanical ventilation in 31 providences in Iran**	278	Cross-sectional survey study	Pt spiritual health (Spiritual Health Questionnaire, SHQ, validated in Muslim populations)	In structural equation modelling, spiritual health (SHQ) was indirectly associated with decreased depression and anxiety [HADS: b=-0.081, p<0.05] via pt-physician communication. Pt spiritual health had positive direct/indirect associated with greater impact of event [IES-R via PP-QOL: b=0.293, p<0.05], and higher post traumatic stress scale [PTSS-14 via PP-QOC: b=0.267, p<0.001]. So though spiritual health is related to less depression/anxiety (indirect), SH was associated with greater PTSD symptoms. Pt spiritual health was not related to QoL directly or indirectly.	Pt anxiety and depression (Hospital Anxiety and Depression Scale, HADS) Quality of communication (Pt-physician and Pt-nurse Quality of Communication Questionnaire, PP-QOL) Impact of Event Scale (scale) Quality of life (Quality of Life in Mechanically Ventilated pts, QoL-MVP)	Low	Cross-sectional study using validated scales for this population; sample size=278; RR=96%; structural equation modeling used to simultaneously assess relationships of multiple variables; generalizability considerations due to population studied (Iranian pts who had been admitted to ICUs in 31 provinces in Iran).	**Method: Locally Developed**
Dadsetan et al. 2021	**Middle East, Iran Adult multiple sclerosis patients recruited from neurology wards of Shahid Bahonar Hospital, affiliated with Kerman University of Medical Sciences in the southeast of Iran**	132	Cross-sectional survey study	Palliative care needs including physical, social, psychological, economic, spiritual dimensions (Instrument developed/validated by the authors)	All patients reported some degree of spiritual needs [μ=31.2, σ=4.1]. Nurses rated patients spiritual needs lower than patients [μ=26.6, σ=3.7, p<0.0001]. Patient also reported physical needs [μ=42.6, σ=9.6], social needs [μ=39.0, SD=6.4], psychological needs [μ=22.3, σ=6.8], and economic needs [μ=14.1, σ=3.0].	Nursing assessment of patient palliative care needs (instrument developed/validated by the authors)	Moderate	Cross-sectional study using validated scales; sample size=132 multiple sclerosis patients from a single hospital site; RR not reported; generalizability considerations due to population studied (multiple sclerosis patients from a single hospital site in Iran).	**Method: Western, modified, translation and cultural context**
Farahani et al. 2019	**Middle East, Iran Cancer MDs and nurses in government-affiliated medical facilities**	136	Cross-sectional survey study	Assessment of spiritual care (SC) perceptions and practices (Spiritual Care Survey, adapted from the Multidimensional Measure of	70.6% of the participants considered spiritual care to be influential in the pts' QOL. However, 64.7% had received no spiritual care training,	General views of spiritual care and provider perspectives on providing different types of spiritual care	Moderate	Descriptive study using validated scales; sample size=136 MDs and nurses in multiple medical facilities in Iran; RR not reported; generalizability	**Method: Western, modified, translation**

				Religiousness and Spirituality)	while 82.4% indicated a willingness to receive training.			considerations due to population studied (oncology MDs and nurses, ~95% Muslim, in government medical facilities in Iran).	
Fawares et al. 2021	**Middle East, Jordan Adult cancer patients receiving palliative care at a single hospital in Jordan**	119	Cross-sectional survey study	Religious beliefs and values (Arabic Beliefs and Values scale)	Patients had high endorsements of religion and spirituality; they had greater endorsement of the spirituality religious beliefs items [3.38±0.33] as compared to the spirituality non-religious beliefs aspect total mean score [2.49±0.50].	Descriptive data only	Moderate	Cross-sectional study using validated scales; sample size=119 cancer patients receiving palliative care at a single hospital in Jordan; RR=70%; generalizability considerations due to population studied (adult cancer patients receiving palliative care a single hospital site in Jordan).	**Method: Western, modified, translation, cultural context, spiritually specific**
Khanjari et al. 2012	**Middle East, Iran Adult cgs of pts with newly diagnosed (within 2-3 weeks) breast cancer recruited from 2 hospitals of Tehran Medical Sciences University l Association. All rig**	150	Prospective cohort study (6 months) ed	Spirituality (Spiritual Perspective Scale, SPS) Religious coping (Brief RCOPE positive and negative: pos RCOPE, neg RCOPE)	Neg rel coping (neg RCOPE) decreased from T1-T2 [p<0.001]. Quality of life (CQOLC) improved from T1-T2 [p<0.001]. In MVA, predictors of increasing QOL T1-2 were decreasing neg RCOPE T1-2 [p<0.001]. Positive rel cope (pos RCOPE) was high and did not change T1-2. No association was found between pos RCOPE and spiritual perspecties with changes in QOL T1-T2.	Cg quality of life (Persion cg Quality of Life Index- Cancer, CQOLC); Cg sense of coherence (Sence of Coherence scale, SOC); Cg health (Health Index, HI)	Moderate	Prospective study using validated scales; sample size=150 cgs from 2 hospitals in Iran; RR=85.7%, including attrition at T2, 65.7%; MVA characterize prospective associations controlling for potential confounding variables; generalizability considerations due to population studied (adult cgs of newly diagnosed breast cancer pts in Iran).	**Method: Western, modified, translation**
Mahdavi et al. 2017	**Middle East, Iran Caregivers of alzheimers dementia pts registered with the Iran Alzheimer's Association**	100	Randomized controlled trial	Intervention: Spiritual group therapy for caregivers, over the course of 5 weeks Control: 5 week support group	The spiritual group therapy mean of the post-test caregiver strain score [32.43±2.73] was significantly lower than pretest [37.16±1.26] [p<0.001]. The mean post-test score of caregiver strain was significantly lower in the intervention group compared to the two other groups [p<0. 001].	Caregiver strain (Caregiver Strain Index, CSI)	Low	RCT of a spiritual care intervention for family cgs (vs. active support group control); sample size=100 cgs of dementia pts in Iran; recruitment rate not reported; generalizability considerations given population studied (cgs of pts with dementia in Iran).	**Method: Western, modified, translation and cultural context**

Rohani et al. 2015	**Middle East, Iran Adult women all stages of breast cancer receiving care at 1 of 2 educational medical facilities in Iran, Tehran**	162	Prospective cohort study (6 months)	Spiritual beliefs and behaviors (Spiritual Perspectives Scale, SPS) Religious coping (RCOPE positive and RCOPE negative) Sense of coherence/meaning to life (Sense of Coherence Scale, SOC)	In MVA, though spiritual beliefs and behaviors (SPS), and religious coping (RCOPE Pos and RCOPE neg) did not predict improvements in QOL (EORTC QLQ-C30) at 6 months, Sense of Coherence (SOC) did predict improvements in QOL at 6 mo [EORTC QLQ: b=0.50, p<0.001].	Quality of life (European Organization for Research and Treatment of Cancer scale, EORTC, QLQ-30)	Low	Prospective study; sample size=499 from the New Haven Established Populations for Epidemiologic Studies of the Elderly, EPESE cohort study population (n=2,812); loss to follow up less than 1%; multivariable regression analysis with adjustment for multiple potential confounders performed to assess prospective relationships of religious/spiritual variables to QOL measures; generalizability considerations due to population studied (adult pts who participated in the EPESE cohort study and died; 80% White; 20% non-White; 49.5% male; from Northeastern US).	**Method: Western, modified, translation**
Yaghoobzadeh et al. 2018	**Middle East, Iran Adult pts with cardiovascular disease hospitalized for greater than 24 hours in a medical institution in Iran**	500	Cross-sectional survey study	Spiritual well-being (Spiritual Well-Being Scale, SWBS)	Mean spiritual well-being (SWBS) was 86.21 (SD 12.46; range 40-116) and mean hope (HHI) was 34.80 (SD 5.05; range 23-46), indicating higher than moderate spiritual well-being and a moderate level of hope. In MVA, hope was a significant predictor of spiritual well-being [b=0.90, 95% CI 0.68-1.13, p<0.001], as were religious belief [b=0.99, 95% CI 0.17-1.80, p=0.018] and female sex [p=0.047]. Spiritual well-being was in turn a significant predictor of hope [b=0.12, 95% CI 0.09-0.16, p<0.001], alongside marital status, educational status and socioeconomic status.	Hope (Herth Hope Index, HHI)	Moderate	Cross-sectional study using validated scales; sample size=500 from a single medical institution in Iran; RR=76.7%; MVA assessed cross-sectional associations; generalizability considerations due to population studied (adult, cardiovascular disease pts admitted to a hospital in Iran).	**Method: Western, modified, translation**

Zafar et al. 2016	**Asia, Pakistan Adult cancer pts undergoing treatment at the tertiary cancer center in Lahore, Pakistan**	520	Cross-sectional survey study	Assessment of religiousness, Preferences regarding EOL care, Pt EOL priorities (survey developed for this study from validated instruments: Multidimensional Health Locus of Control scales, Singer et al. EOL care items, survey piloted)	96% important, 4% religion not important. Average rank order preferences of priorities at EOL: (1) spiritual/religious well-being; (2) not being a burden to others; (3) strengthening relationships with family/friends; (4) receiving adequate pain control and symptoms management; (5) achieving a sense of control and dignity; (6) avoiding inappropriate prolongation of dying.	Description of pt religiousness and EOL priorities	Moderate	Cross-sectional study using validated scales; sample size=520 cancer pts from a single medical center in Pakistan; RR=98.6%; MVA assess how religion/spirituality relates to EOL advance care planning, with control for proxy measures; generalizability considerations due to population studied (cancer pts, 58.1% female, average age 47.8, undergoing active cancer treatment at a single center in Pakistan).	**Method: Locally Developed**
Gravier et al. 2020	**Mixed, Brazil, Chile, India, Jordan, US Adult advanced cancer pts recruited from 5 outpatient palliative care clinics in Brazil, Chile, India, Jordan, USA**	728	Cross-sectional survey study	Edmonton Symptom Assessment Scale (ESAS), including ESAS Spiritual Pain (ESAS-SP) and Financial Distress (ESAS- FD) items	Meaning in life (MILS) did not differ between countries [p=0.11]. In MVA, spiritual pain (ESAS- SP) was associated with worse meaning in life [b=-0.19, p<0.001], depression was associated with worse MIL [b=-0.26, p<0.001], and financial impact of illness was associated with worse MIL [b=-0.16, p<0.001]. Optimism was associated with greater MIL [b=0.33, p<0.001].	Meaning in Life Scale (MILS)	Moderate	Cross-sectional study using validated scales as a planned secondary analysis of a prospective trial; sample size=728 from 5 medical centers in 5 countries; RR not reported; MVA control for multiple potential confounds to characterize cross-sectional associations; generalizability considerations due to population studied (adult advanced cancer pts receiving palliative care in Brazil, Chile, India, Jordan, USA).	**Method: Western, modified, translation**
Kang et al. 2019	**Mixed, Canada, Korea, Hong Kong, Iran, US RCTs and other intervention studies of meaning centered interventions in advanced cancer pts**	623	Meta-analysis	Meaning-centered interventions, including: meaning-centered psychotherapy, logotherapy, or meaning-making interventions	10 studies included in the meta-analysis (6 randomized, 4 non-randomized) with interventions being meaning-centered psychotherapy, logotherapy, or meaning making interventions. Studies categorized by outcomes assessed: meaning-in-life (6 studies) [effect size=-0.96 (95% CI: -1.28, -0.64), p<0.001]; spiritual well-	Effect sizes by well-being outcomes, categorized as: meaning in life, spiritual well-being, QOL, anxiety, physical symptoms	Low	Meta-analysis of randomized controlled trial and other interventions of meaning centered interventions using validated scales; sample size=10 studies, 623 cancer pts; generalizability considerations given populations studied (cancer pts, all stages from 6 countries).	--

					being (3 studies) [effect size=-0.37 (95% CI: -0.61, -0.13), p=0.002]; QOL (6 studies) [effect size=-0.48 (95% CI: -0.67, -0.28), p<0.001]; anxiety (3 studies) [effect size=-0.28, (95% CI: -0.52, -0.04), p=0.02]; physical symptoms (3 studies) [effect size=-0.31 (95% CI: -0.56, -0.05), p<0.001].				
Mah et al. 2019	**Mixed, Canada, Kenya Pts who died in 3 Kenyan hospices [N=127], compared to pts who died of advanced cancer in Ontario, Canada [N=602]**	729	Cross-sectional survey study	Patient quality of death and dying as assessed by the family caregiver (The Quality of Death and Dying Questionnaire, QODD), with 6 domains: Symptoms and Personal Care, Preparation for Death, Moment of Death, Family, Treatment Preferences, and Whole Person Concerns	As compared to the Canadian sample, quality of death outcomes (QODD) in the Kenyan sample were worse on 14 quality of dying and death concerns and on overall quality of dying and death [all p<0.001] but better on five concerns, including interpersonal and religious/spiritual concerns [all p<0.005]. In MVA, overall quality of dying was associated with better pt care experiences with symptom s and personal care, interpersonal care, and of religious/spiritual concerns [all p<0.01].	Description of quality of death and dying, with comparisons between the Canadian and Kenyan samples	Moderate	Cross-sectional study using validated scales; sample size=729 from multiple care settings in 2 countries, Canada and Kenya; RR not reported; cgs provided proxy reports on pt quality of death in last week of life; MVA to assess how individual quality of death any dying concerns relate to overall quality of death; generalizability considerations due to population studied (cgs of pts who died in hospices in Kenya and Canada).	**Method: Western, modified, translation and cultural context**
Malhotra et al. 2020	**Asia, China, India, Myanmar, Sri Lanka, Vietnam Adult advanced cancer patients recruited from 7 hospitals in 5 countries (China, India, Myanmar, Sri Lanka and Vietnam)**	1,378	Cross-sectional survey study	Spiritual suffering (reverse scaling of the meaning/peace domain of the spiritual well-being scale to function as as 'spiritual suffering scale; Functional Assessment of Chronic Illness Therapy - FACIT- Sp)	Among advanced cancer patients, "Spiritual suffering" (reversed meaning/peace FACIT-Sp) is less among those with more education [b=-0.26, SE=0.12, p<0.05], is greater among those of lower socioeconomic status [b=7.57, SE=1.66, p<0.01], and is greater in Sri Lanka [b=16.55, SE=2.04, p<0. 01], India [b=11.53, SE=1.89, p<0.01], and Vietnam [b=5.81, SE=2.02, p<0.01] as compared to China.	Physical and functional suffering (physical and functional well-being sub-scales of the Functional Assessment of Cancer Therapy - General, FACT-G version 4) Pain (Visual Analogue Pain Scale) Psychological suffering (Hospital Anxiety and Depression Scale, HADS) Social suffering(social	Moderate	Cross-sectional study using validated scales (using a domain of the scale, FACIT-Sp meaning/peace, reversed to assess spiritual suffering); sample size=1387 advanced cancer patients from 7 hospitals in 5 Asian countries; RR=90%; MVA assessed predictors of spiritual suffering; generalizability considerations due to population studied (adult advanced cancer patients recruited from 7 hospital	**Method: Western, modified, translation**

						subscale of the FACT-G)		settings in 5 countries: China, India, Myanmar, Sri Lanka).	
Chacko et al. 2014	**Asia, India Pts with stage III or IV cancer being seen at a single medical center in Vellore, India**	140	Cross-sectional survey study	Survey assessing importance of physical, emotional, social, and spiritual dimensions of EOL care (modified EOL survey from Davidson, 2019)	Addressing spiritual needs was moderately to very important to 96.4%. "Extremely important" aspects of spiritual care included: being able to pray [89.3%], others praying for the patient [86%], meeting spiritual advisors [63.6%], discussing spiritual issues [60.7%], and considering spiritual preferences while making decisions about life prolonging therapies [59.3%]. Health professionals rated the same items markedly lower [52.5%, 42.5%, 42.5%, 50% and 35% respectively]. Ideal person for addressing spiritual needs: family/friends [38.6%], spiritual advisor [35.7%], others [17.8%], nurse [5%], MD [2.9%].	Description of pt perceptions of spiritual dimensions of EOL care	Moderate	Cross-sectional study with validated survey; sample size=140 pts from a single medical center in India; RR=82.8%, generalizability considerations due to population studied (advanced, cancer pts in India, 51.4% female. 69.3% Hindu, 20.0% Christian, 9.3% Muslim, 1.4% other).	**Method: Western, modified, translation, cultural context, spiritually specific**
Haokip et al. 2022	**Asia, India Adult cancer patients recruited from a single, large government hospital, All India Institute of Medical Sciences, Rishikesh, India**	103	Cross-sectional survey study	Spiritual beliefs (System of Belief Inventory, SBI- 15R)	Most (77%) considered themselves moderately or very spiritual. In univariable analysis, spirituality was inversely associated with depression [r=-0.209, p=0.05].	Depression symptoms (Patient Health Questionnaire Hindi version, PHQ-9)	Moderate for descriptive data; Severe for associations with depression	Cross-sectional study using validated scales; sample size=103 cancer patients from a single hospital in India; RR=93%; univariable analysis examined associations of spirituality with depressive symptoms; generalizability considerations due to populations studied (cancer patients recruited from a single hospital in India).	**Method: Western, modified, translation, cultural context, spiritually specific**
Mishra et al. 2022	**Asia, India Adult cancer inpatients and outpatients, and their informal caregivers (when present) seen by oncology and**	500	Cross-sectional survey study ed	Spiritual suffering during the COVID-19 pandemic questionnaire (3-item questionnaire developed/validated by the authors)	53.6% of patients reported lack of spiritual clarity and hope; among caregivers, 20% reported suffering a lack of spiritual clarity and hope. 35% of patients indicated challenges from a lack of	Other domains of suffering during cancer care in the COVID-19 pandemic were examined: physical, logistic (including treatment delays)	Moderate	Cross-sectional study using validated scales; sample size=500 patients and 360 caregivers from a single hospital setting in New Delhi, India; RR=97%; generalizability	**Method: Unclear**

	**palliative care services during the COVID-19 pandemic at a single hospital in New-Delhi, l AssocIniadtiaion. All rig**				religious practice during the pandemic; 30% of caregivers indicated a lack of religious practice during the pandemic.	psychological, and socioeconomic		considerations due to population studied (adult cancer patients and their informal caregivers receiving treatment at a single hospital in New Delhi, India).	
Satija et al. 2022	**Asia, India Adult advanced cancer patients who were recruited from oncology and palliative medicine clinics at a tertiary cancer hospital in India**	200	Cross-sectional survey study	Spiritual Well-being (FACIT-Sp)	73% were not aware of the stage of their malignancy. In MVA, patients who were aware of their advanced cancer stage had worse FACIT-Sp faith subdomain scores [b=-1.6, (95% CI: -3.1, -0.1, p=0.03] (alternate numerical interpretation is those with greater spiritual well-being have less awareness of their cancer).	Awareness of stage of malignancy	Moderate	Cross-sectional survey study using validated scales from multiple clinics at a single hospital setting in India; RR=93%; MVA assessed relationship of spiritual well-being to awareness of cancer stage; generalizability considerations due to population studied (adult, metastatic cancer patients from oncology and palliative care clinics at a single tertiary hospital in India).	**Method: Western, modified, translation**
Akuoko et al. 2022	**Sub-Saharan Africa, Ghana Adult female advanced breast cancer outpatients from two outpatient clinics in Kumasi, Ghana**	176	Cross-sectional survey study	Spiritual needs (Spiritual Need Assessment for Patients, SNAP)	Most [92.1%] had at least one spiritual need (SNAP). Needs included: 92.6%, someone to bring you spiritual texts; 92.1%, finding peace of mind; 90.9%, finding forgiveness; 90.3%, your relationship with God or something beyond yourself; 90.3%, visits from clergy/imam of your own faith community; 90.3%, religious rituals such as chant, prayer, lighting candles or incense, anointing, communion; 88.6%, finding hope; 87.5%, meaning and purpose of human life; 84.7%, personal meditation or prayer practices. Older women reported greater religious needs compared to younger women	Supportive Care Needs Survey-Long Form; modified Client Service Receipt Inventory	Low	Cross-sectional study using validated scales; sample size=176 from two outpatient clinics in Kumasi, Ghana; RR=93%; generalizability considerations due to population studied (female, advanced breast cancer patients from two clinics in Kumasi, Ghana).	**Method: Western, modified, spirituality specific**
Lowther et al. 2018	**Sub-Saharan Africa, Kenya Adults HIV pts, on**	120	Randomized controlled trial	Intervention: 5 visits to a palliative care nurse who provided assessment and care	A RCT of a nurse-led palliative care intervention for pts with HIV found in	Care received (Client Services Receipt Inventory, CSRI);	Moderate	RCT using validated scales (control was usual care); sample size=120 pts with	**Method: Western, modified,**

	**ART for at least 1 month, with a pain or symptom score of 3 (from a possible range of 0-best to 5-worst) on the African Palliative Care Association Palliative Outcome Scale (APOS), being seen at an HIV clinic in Mombasa**			plan for addressing physical, psychological social and spiritual problems. Complex pts were referred to specialty palliative care Controls: Usual HIV care (monthly clinic appointments) vs. usual care (1:1)	assessments of 22 components of the palliative care intervention (medication and psychosocial), discussion about spiritual worries was higher in the intervention arm vs usual care [95% vs. 57%, p<0.001], emotional support from staff [100% vs. 80%, p<0. 001], use of weak opioids [60% vs. 18%, p<0.001], discussion about the future [67% vs. 22%, p<0.01] constipation medication [27% vs. 12%; p=0.04], support for the family in planning for the future [65% vs. 22%, p<0.001]. Mental well-being had greater numeric improvements in intervention arm vs. usual care [57% vs. 52%, but no analyses reported].	Qualitative exit interviews to further assess pt perceptions of the intervention; Mental health (MOS-HIV)		HIV and active symptoms; MOS-HIV changes not analyzed (likely non-significant); recruitment rate of eligible pts=44.3%; generalizability considerations given population studied (HIV+ pts seen at an HIV clinic in Mombasa, Kenya).	**translation, unstructured interview**
Ndiok & Ncama 2018	**Sub-Saharan Africa, Nigeria Adult cancer pts seen at 2 teaching hospitals in Nigeria, cgs could respond on behalf of cognitively impaired pts**	455	Cross-sectional survey study	Pt spiritual and existential issues (study developed scale to assess pt spiritual/existential issues for their population, with literature review, pilot testing, and validation)	Existential/spiritual needs were reported by 49.7% to 71.8%, including 71.8% difficulties with acceptance; 63.6% difficulties in feeling useful; 55.7% difficulties in being available to others; 55.0% difficulties concerning the meaning of death, 49.7% difficulties in confidence in God/religion.	Description of spiritual needs and existential issues	Low	Cross-sectional study using validated scales; sample size=455 from two medical institutions; RR=82%; generalizability considerations due to population studied (adult advanced cancer pts from two hospitals in Nigeria).	**Method: Locally Developed**
Nkhoma et al. 2018	**Sub-Saharan Africa, Kenya Adult, HIV/AIDS pts recruited from an outpatient clinic in Mombasa, Kenya**	400	Cross-sectional survey study	Existential/spiritual concerns (African Palliative Outcomes Scale, APOS)	36.8% reported being in lowest 3 categories of peace (APOS), 23.5% in lowest 3 categories of life worthwhile. In MVA, decreasing age associated with greater existential/spiritual concerns [p=0.001], as was higher CD4 count [p=0.014], and TB treatment [p=0.02].	Description of existential and spiritual concerns	Moderate	Cross-sectional study using validated scales; sample size=400 from one medical institution; RR=84.5%; generalizability considerations due to population studied (adult HIV/AIDS pts from one region in Kenya).	**Method: Western, modified, translation**

Ratshikana-Moloko et al. 2020	**Sub-Saharan Africa, South Africa Adult advanced cancer pts recruited from the Gauteng Center for Palliative Care in Soweto, South Africa**	233	Prospective cohort study	Pt reports of religious/spiritual needs and care (Spiritual Needs and Care questionnaire); Religious/spiritual characteristics (Coping with Cancer study items)	Most pts were religious or spiritual [94.8%]. Most reported having spiritual needs [97.8%], with the most common being seeking a closer connection to God [92.6%] and forgiveness of sins [90.8%]. 39.5% of pts received spiritual care. Pts receiving spiritual care had less pain [2.82±1.23 vs. 1.93±1.69; higher POS scores indicate less pain] and were more likely to die at home [57.5% vs. 33.7%]. In MVA, spiritual care associated with reduced pain [POS: OR=0.33 (95% CI: 0.11, 0.95)] and increased family worry [POS: OR=3.43 (95% CI: 1.10, 10.70)].	Description of pt spiritual needs and care; Pt palliative care outcomes (Palliative Care Outcomes scale, POS); Pt location of death	Moderate	Prospective cohort study using validated scales; sample size=233 advanced cancer pts from a single medical institution in South Africa; RR=72.5%; multivariable models assessed prospective associations with control for baseline proxy measures and multiple other potential confounding factors; generalizability limitations due to population studied (advanced cancer pts in South Africa).	**Method: Western, modified, spirituality specific**
Selman et al. 2011	**Sub-Saharan Africa, South Africa, Uganda Adult cancer, HIV, and other advanced illness pts seen at 4 palliative care facilities in South Africa and 1 hospice in Uganda**	285	Cross-sectional survey study	Quality of life (Missoula Vitas Quality of Life Index, QVQOLI): subscales symptoms, function, interpersonal, well-being, transcendent; assessment of what is important to pts using QVQOLI	Pts assessed what is most important to their QOL, which were: close relationships [mean=4.13], feeling at peace [mean=4.12], sense of meaning in life [mean=4.10], being active [mean=3.84], physical comfort [mean=2.58]. The transcendent subscale was most highly correlated with pt global QOL [r=0.77, p<0.001], as compared to other domains of QOL.	Description of pt quality of life and description of what pts deem as most important to quality of life	Low	Cross-sectional study using validated scales; sample size=285 from 4 palliative care sites in South Africa and 1 hospice in Uganda; RR=100%; study findings largely descriptive (MVA not used for assessment of associations with pt QOL and this finding not considered "low"); generalizability considerations due to population studied (adult HIV and other advanced illnesses receiving palliative and/or hospice care in 5 care centers in South Africa and Uganda).	**Method: Western, modified, translation**
Weru et al. 2020	**Sub-Saharan Africa, Kenya Adult advanced cancer (stage 3 and 4) patients receiving care at clinical settings of the Aga**	144	Randomized trial (6 weeks)	Intervention: Dignity therapy Control: Treatment as usual	There was no significant difference between ESAS Quality of life in the intervention versus the control group.	Quality of Life (Edmonton Symptom Assessment Scale, ESAS)	Moderate	Randomized trial of dignity intervention versus usual care among patients with advanced cancers seen at a single university hospital in Nairobi, Kenya; Consent Rate=89%; generalizability	**Method: Western, unmodified. Intervention: Western, unmodified**

	**Khan University hospital Nairobi**							considerations given the population studied (adult, advanced cancer patients a single university hospital setting in Nairobi, Kenya).	

Supplementary Table S3: Caregiver data

Reference	Religious tradition	Population	Sample size	Design	Spiritual care measures	Method of measures	Outcome variables	Summary of results	Bias rating	Bias description
Demirbag et al. 2018	Muslim	**Middle East, Turkey Nurses who worked at five hospitals in the Eastern Black Sea Region of Turkey**	1,254	Cross-sectional survey study	Characteristics of spiritual care provision (questionnaire developed for study based on literature review)	**Method: Locally Developed**	Spiritual care interventions provided by nurses	91% nurses never ask about pt spiritual needs, 98.1% had never received spiritual care training. Training was a strong predictor of assessing spiritual needs [X^2=19.96, p<0.01].	Moderate	Cross-sectional study using a validated scale assessing spiritual care perceptions/practices; sample size=1254 from 5 medical institutions; RR=71%; findings largely descriptive, UVA to characterize predictors of spiritual care; generalizability considerations due to population studied (nurses, 100% women, 100% Muslim, from the Eastern Black Sea region of Turkey.
Khanjari et al. 2012	Muslim*	**Middle East, Iran Adult cgs of pts with newly diagnosed (within 2-3 weeks) breast cancer recruited from 2 hospitals of Tehran Medical Sciences University l Association.**	150	Prospective cohort study (6 months)	Spirituality (Spiritual Perspective Scale, SPS) Religious coping (Brief RCOPE positive and negative: pos RCOPE, neg RCOPE)	**Method: Western, modified, translation**	Cg quality of life (Persion cg Quality of Life Index- Cancer, CQOLC); Cg sense of coherence (Sence of Coherence scale, SOC); Cg health (Health Index, HI)	Neg rel coping (neg RCOPE) decreased from T1-T2 [p<0.001]. Quality of life (CQOLC) improved from T1-T2 [p<0.001]. In MVA, predictors of increasing QOL T1-2 were decreasing neg RCOPE T1-2 [p<0.001]. Positive rel cope (pos RCOPE) was high and did not change T1-2. No association was found between pos RCOPE and spiritual perspecties with changes in QOL T1-T2.	Moderate	Prospective study using validated scales; sample size=150 cgs from 2 hospitals in Iran; RR=85.7%, including attrition at T2, 65.7%; MVA characterize prospective associations controlling for potential confounding variables; generalizability considerations due to population studied (adult cgs of newly diagnosed breast cancer pts in Iran).
Vigna et al. 2020	Catholic; Spiritualist; Evangelical	**Latin America, Brazil Adult informal caregivers of seriously ill patients receiving palliative care at a the Hospital da Luz, in São Paulo, Brazil**	178	Cross-sectional survey study	Spirituality, religiousness and personal beliefs (WHO Quality of life - Spirituality, religiousness and personal beliefs, WHOQOL-SRPB, scale)	**Method: Western, modified, translation**	Psychological morbidity (Self-Reporting Questionnaire, SRQ-20) Caregiver burden (Zarit Burden Interview, ZBI) Quality of life (WHOQOL)	Higher spirituality as measured by the SRPB score was associated with lower Zarit scores [r=-0.45, p=0.001] and also with lower SQR-20 score [r=-0.37, p<0.001]; In MVA, spirituality predicted for less burden [b=-0.53, p<0.001].	Moderate	Cross-sectional survey study of caregivers of seriously ill patients receiving palliative care at a single hospital in Brazil; RR=96%; MVA employed to assess relationship of psychological well-being measure with spiritual well being, adjusting for potential confounding factors.

Only three studies focused on family caregivers. Among Iranian caregivers of women with breast cancer, a lower sense of coherence and greater use of negative religious coping predicted poorer quality of life at six months (Khanjari et al., 2012). In Brazil, a study of 178 family caregivers in palliative care found that higher spirituality, particularly inner peace and meaning, was the strongest protective factor against caregiver burden. In comparison, lower spirituality and psychiatric distress were correlated with greater emotional strain (Vigna et al., 2020). A separate Iranian trial of spiritual group therapy for Alzheimer's caregivers significantly reduced caregiver strain ($p<0.001$; Mahdavi et al., 2017)

Supplementary Table S4: Measurement tools used in included studies

Measure (construct)	Spiritual focus	LMIC use in review — all included studies	Assessment	Adaptation class
FACIT-Sp / FACIT-Sp-12 — Functional Assessment of Chronic Illness Therapy–Spiritual Well-Being	Primary	China: Liu (2022); Yang (2021); Wang (2021); Xiao (2022). India: Satija (2022). Multi (China/India/Sri Lanka/Vietnam/Myanmar): Malhotra (2020; APPROACH)	HIC Developed	Translated
Brief Religious Coping Scale (Brief RCOPE)	Primary	Brazil: Ramirez (2012). Iran: Rohani (2015); Iran (caregivers): Khanjari (2012). South Africa: Ratshikana-Moloko (2020; selected items)	HIC Developed	Translated, Cultural, Spiritual
System of Belief Inventory (SBI-15R)	Primary	India: Haokip (2022)	HIC Developed	Translated
Beliefs & Values Scale (BVS)	Primary	Jordan: Fawares (2021)	HIC Developed	Translated, Cultural, Spiritual
Muslim/Iranian Spiritual Health Questionnaire (MSHQ/SHQ)	Primary	Iran: Bashar (2018)	Locally Developed (LMIC)	—
Spiritual Needs Assessment for Patients (SNAP)	Primary	Ghana: Akuoko (2022)	HIC Developed	Translated, Cultural, Spiritual
Thai Spiritual Needs Scale (TSNS)	Primary	Thailand: Wisesrith (2021)	Locally Developed (LMIC)	—
Spiritual Care Requirements Scale (SCRS)	Primary	Turkey: Şahan & Yıldız (2022)	Locally Developed (LMIC)	—
Spiritual Perspectives Scale (SPS)	Primary	Iran: Rohani (2015); Iran (caregivers): Khanjari (2012)	HIC Developed	Translated
Spiritual Care Survey (RSCC/MMRS-adapted) — SC practices/perceptions	Primary	Middle East (14 countries): Bar-Sela (2019; SCC & PSC). Iran: Farahani (2019)	HIC Developed	Translated, Cultural
"Spiritual Needs & Care" questionnaire (Coping-with-Cancer–derived)	Primary	South Africa: Ratshikana-Moloko (2020)	Locally Developed (LMIC)	—

Iranian Sources-of-Hope questionnaire	Primary	Iran: Afrooz (2014)	Locally Developed (LMIC)	—
Nigerian Spiritual/Existential Issues Scale (NSEI)	Primary	Nigeria: Ndiok & Ncama (2018)	Locally Developed (LMIC)	—
Prayer-for-Health (PFH) items	Primary	Malaysia: Chui (2014)	Locally Developed (LMIC)	—
Spiritual Suffering (SSS-COVID) items	Primary	India: Mishra (2022)	Locally Developed (LMIC)	—
Nurse Spiritual-Care Interventions Questionnaire	Primary	Turkey: Demirbag & Ozkan (2018)	Locally Developed (LMIC)	—
**Measure (construct)**	**Spiritual focus**	**LMIC use in review — all included studies**	**Assessment**	**Adaptation class**
Palliative care Outcome Scale (POS), generic version	Subdomain/item used (existential/spiritual: self-worth)	Hong Kong/Taiwan/Thailand: Kwok (2021)	HIC Developed	Translated
APCA African Palliative Outcome Scale (APCA African POS), developed by the African Palliative Care Association	Subdomain/item used (existential/spiritual: “peace”, “life worthwhile”)	Kenya: Nkhoma (2018); Lowther (2018). South Africa: Ratshikana-Moloko (2020)	HIC Developed	Translated, Cultural
POS-Symptoms for Parkinson’s Disease (POS-S PD)	None	Hong Kong/Taiwan/Thailand: Kwok (2021)	HIC Developed	Translated
Missoula-VITAS Quality of Life Index (MVQOLI)	Subdomain/item used (Transcendent; “feeling at peace” in Well-being)	South Africa & Uganda: Selman (2011)	HIC Developed	Translated
Edmonton Symptom Assessment System (ESAS)	Subdomain/item used (Spiritual Pain in some cohorts)	Brazil/Chile/India/Jordan: Gravier (2020). Kenya: Weru (2020)	HIC Developed	Translated
EORTC QLQ-C30 — EORTC Quality of Life Questionnaire-C30	None	China: Chen (2021). Iran: Rohani (2015)	HIC Developed	Translated
WHOQOL-BREF — WHO Quality of Life-BREF	None	Brazil: Ramirez (2012); Vigna (2020); Camargos (2015)	HIC Developed	Cross-cultural/general
FACT-G (v4) — Functional Assessment of Cancer Therapy–General	None	Multi (China/India/Sri Lanka/Vietnam/Myanmar): Malhotra (2020; APPROACH)	HIC Developed	Translated
Hospital Anxiety and Depression Scale (HADS)	None	Iran: Bashar (2018). China: Chen (2021). Multi: Malhotra (2020). Brazil: Ramirez (2012). Brazil: Valentino (2022)	HIC Developed	Translated
General Health Questionnaire-60 (GHQ-60)	None	Brazil: Martínez & Custódio (2014)	HIC Developed	Translated
Patient Health Questionnaire-9 (PHQ-9)	None	India: Haokip (2022). China: Xiao (2022)	HIC Developed	Translated
Zung Self-Rating Anxiety Scale / Zung Self-Rating Depression Scale (SAS / SDS)	None	China: Wang (2021)	HIC Developed	Translated

Family Adaptability and Cohesion Evaluation Scales II (FACES-II)	None	China: Wang (2021)	HIC Developed	Translated
Patient Dignity Inventory (PDI)	None (existential, not labelled spiritual)	China: Xiao (2022)	HIC Developed	Translated
EuroQol 5-Dimension (EQ-5D)	None	Hong Kong/Taiwan/Thailand: Kwok (2021)	HIC Developed	Translated
Medical Outcomes Study HIV Health Survey (MOS-HIV)	None	Kenya: Lowther (2018)	HIC Developed	Translated
Visual Analogue Scale for Pain (VAS Pain)	None	Multi: Malhotra (2020)	HIC Developed	Translated
Numerical Rating Scale (NRS) for Pain	None	China: Yang (2021)	HIC Developed	Translated
Meaning in Life Scale, Short Form (MILS-SF)	None (meaning/existential, not labelled spiritual)	Brazil/Chile/India/Jordan: Gravier (2020)	HIC Developed	Translated
Life Orientation Test–Revised (LOT-R)	None	China: Yang (2021)	HIC Developed	Translated
Problems and Needs in Palliative Care — short version (PNPC-sv)	Subdomain/item used (Spiritual domain)	Indonesia: Effendy (2015)	HIC Developed	Translated
Caregiver Quality of Life Index–Cancer (CQOLC)	None	Iran: Khanjari (2012)	HIC Developed	Translated
Sense of Coherence (SOC)	None	Iran: Rohani (2015)	HIC Developed	Translated
Health Index (self-rated health)	None	Iran: Khanjari (2012)	HIC Developed	Modification unclear
Templer's Death Anxiety Scale (T-DAS)	None	Iran: Amini (2020)	HIC Developed	Intervention, Local
Death Anxiety Scale	None	Turkey: Şahan & Yıldız (2022)	HIC Developed	Modification unclear
Quality of Dying and Death (QODD)	Subdomain/item used (Religious/Spiritual concerns)	Kenya: Mah (2019)	HIC Developed	Translated, Cultural
Supportive Care Needs Survey, Long Form (SCNS-LF)	Subdomain/item used (Spiritual needs domain)	Ghana: Akuoko (2022)	HIC Developed	Translated, Cultural
Client Service Receipt Inventory (CSRI)	None	Ghana: Akuoko (2022); Kenya: Lowther (2018)	HIC Developed	Translated, Cultural
Structural-Functional Social Support Scale (SFSS)	None	Myanmar: Myint (2021)	HIC Developed	Translated
Complementary and Alternative Medicine Use questionnaire (CAM-Use)	None (includes prayer behaviour, not a spiritual subdomain)	Malaysia: Chui (2014)	Locally Developed (LMIC)	—
Quality of Communication (QOC) — patient–physician/patient–nurse	None	Iran: Bashar (2018)	HIC Developed	Modification unclear

Post-Traumatic Stress Symptoms-14 (PTSS-14)	None	Iran: Bashar (2018)	HIC Developed	Modification unclear
ICU Memory Tool (ICU-MT)	None	Iran: Bashar (2018)	HIC Developed	Modification unclear
Impact of Event Scale–Revised (IES-R)	None	Iran: Bashar (2018)	HIC Developed	Translated
Quality of Life in Mechanically Ventilated Patients (QoL-MVP)	None	Iran: Bashar (2018)	HIC Developed	Translated
Palliative Care Assessment Form (PCAF) — PC effectiveness	Subdomain/item used (includes spiritual effectiveness/needs)	Thailand: Chaiviboontham (2015)	Locally Developed (LMIC)	—
Iranian Palliative-Care Needs Scale (IPCN) (incl. spiritual)	Subdomain/item used (Spiritual needs domain)	Iran: Dadsetan (2021)	Locally Developed (LMIC)	—
Palliative Care Assessment Tool (PCAT, Myanmar) (incl. desire for spiritual support)	Subdomain/item used (Desire for spiritual support)	Myanmar: Myint (2021)	Locally Developed (LMIC)	—
End-of-Life care survey (Davison-derived) — EoL preferences & importance	Subdomain/item used (spiritual importance/preferences)	India: Chacko (2014)	HIC Developed	Translated, Cultural
End-of-Life Factors of Importance & Preferred Place of Death (EoL-FI / PPoD) (study-developed)	Subdomain/item used (spirituality as EoL factor)	Brazil: Valentino (2022)	Locally Developed (LMIC)	—
End-of-Life Priorities (Singer) / MHLC / GSE (composite)	Subdomain/item used (spiritual well-being in EoL priorities)	Pakistan: Zafar (2016)	HIC Developed	Translated, Cultural
Caregiver Strain Index (CSI)	None	Iran: Mahdavi (2017)	HIC Developed	Translated
Self-Reporting Questionnaire-20 (SRQ-20)	None	Brazil: Vigna (2020)	HIC Developed	Translated
Zarit Burden Interview (ZBI)	None	Brazil: Vigna (2020)	HIC Developed	Translated
Herth Hope Index (HHI)	None	China: Wang (2021); Iran: Yaghoobzadeh (2018)	HIC Developed	Translated
